# A Highly Efficient Phosphorescence/Fluorescence Supramolecular Switch Based on a Bromoisoquinoline Cascaded Assembly in Aqueous Solution

**DOI:** 10.1002/advs.202200524

**Published:** 2022-03-13

**Authors:** Xian‐Yin Dai, Yu‐Yang Hu, Yonghui Sun, Man Huo, Xiaoyun Dong, Yu Liu

**Affiliations:** ^1^ College of Chemistry State Key Laboratory of Elemento‐Organic Chemistry Nankai University Tianjin 300071 P. R. China

**Keywords:** delayed fluorescence, phosphorescence energy transfer, photoswitch, room‐temperature phosphorescence, supramolecular assembly

## Abstract

Despite ongoing research into photocontrolled supramolecular switches, reversible photoswitching between room‐temperature phosphorescence (RTP) and delayed fluorescence is rare in the aqueous phase. Herein, an efficient RTP‐fluorescence switch based on a cascaded supramolecular assembly is reported, which is constructed using a 6‐bromoisoquinoline derivative (G_3_), cucurbit[7]uril (CB[7]), sulfonatocalix[4]arene (SC4A4), and a photochromic spiropyran (SP) derivative. Benefiting from the confinement effect of CB[7], initial complexation with CB[7] arouses an emerging RTP signal at 540 nm for G_3_. This structure subsequently coassembles with amphiphilic SC4A4 to form tight spherical nanoparticles, thereby further facilitating RTP emission (≈12 times) in addition to a prolonged lifetime (i.e., 1.80 ms c.f., 50.1 µs). Interestingly, following cascaded assembly with a photocontrolled energy acceptor (i.e., SP), the efficient light‐driven RTP energy transfer occurs when SP is transformed to its fluorescent merocyanine (MC) state. Ultimately, this endows the final system with an excellent RTP–fluorescence photoswitching property accompanied by multicolor tunable long‐lived emission. Moreover, this switching process can be reversibly modulated over multiple cycles under alternating UV and visible photoirradiation. Finally, the prepared switch is successfully applied to photocontrolled multicolor cell labeling to offer a new approach for the design and fabrication of novel advanced light‐responsive RTP materials in aqueous environments.

## Graphical abstract

A highly reversible supramolecular photoswitch between room‐temperature phosphorescence (RTP) and delayed fluorescence is constructed. This photoswitch is based on a bromoisoquinoline cascaded assembly and is successfully prepared in the aqueous phase. It benefits from light‐driven supramolecular RTP energy transfer to exhibit multicolor tunable long‐lived emission, and is successfully applied to photocontrolled multicolor cell labeling.

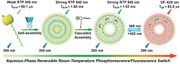

## 1 Introduction

Supramolecular photoswitchable luminescent assemblies have evolved as a compelling research focus owing to their noninvasive and controllable features, which render them ideal candidates for diverse applications in biological systems and materials science.^[^ ^1^ ^]^ Compared with fluorescent assemblies, room‐temperature phosphorescence (RTP)‐based assemblies have attracted the attention of chemists owing to their characteristic superiorities, including their large Stokes shifts and long lifetimes, which have allowed their application in various fields, such as security protection,^[^ ^2^ ^]^ optical sensors,^[^ ^3^ ^]^ and bioimaging.^[^ ^4^ ^]^ To date, a number of delicate approaches have been developed to obtain purely organic RTP emission, such as crystal formation,^[^ ^5^ ^]^ polymerization,^[^ ^6^ ^]^ halogen and hydrogen bonding,^[^ ^7^ ^]^ and doping into rigid polymer matrices.^[^ ^8^ ^]^ More specifically, host–guest complexation mediated by cucurbiturils has recently emerged as an alternative and practicable strategy for the construction of RTP systems in both the solid and the solution phase.^[^ ^9^ ^]^ Such tight encapsulation of guest molecules by the cucurbiturils can not only significantly enhance the intersystem crossing process and shield quenchers from the liquid medium, but it can also concurrently restrain the nonradiative relaxation of the triplet state, thereby greatly improving the RTP performance.^[^ ^10^ ^]^ Notably, a multivalent assembly strategy that incorporates multiple interactions, such as hydrogen bonding, hydrophobic interactions, and electrostatic interactions, has been developed as a synergistic enhancement method to achieve efficient supramolecular RTP emission, especially in aqueous solutions.^[^ ^11^ ^]^

More recently, supramolecular RTP energy transfer systems based on transfer from phosphors to commercial fluorescent acceptors have invoked growing interest due to their ability to endow the purely organic chromophores with long‐lived singlet emission via a delayed sensitization process under ambient conditions.^[^ ^12^ ^]^ This, in turn, leads to multicolor delayed fluorescence emission that could be applicable in areas such as biological time‐resolved imaging,^[^ ^13^ ^]^ anti‐counterfeiting, and information encryption.^[^ ^14^ ^]^ Impressively, Kuila and George reported an efficient organic afterglow system based on phosphorescence energy transfer from a coronene tetracarboxylate salt to highly fluorescent dyes by embedding the dyes into an amorphous poly(vinylalcohol).^[^ ^15^ ^]^ In addition, the construction of stimuli‐responsive RTP energy transfer systems is a new development tendency and provides novel methods for the manipulation of tunable emissions in a controlled manner, wherein the use of light as a stimulus is of particular interest due to its clean, noninvasive, and easily controlled nature.^[^ ^16^ ^]^ For example, Xu and coworkers constructed a fluorescence–RTP photoswitch that acted in both the powder form and in a doped polymer film, where the photoswitch was based on intramolecular light‐controllable photocyclization and decyclization processes.^[^ ^17^ ^]^ In addition, Ma et al. reported a general approach to remotely regulate persistent luminescence in solids and DMF solutions by manipulating the isomerization of the energy acceptor.^[^ ^18^ ^]^ Furthermore, Sessler and coworkers described a supramolecular network grafted with pillar[5]arene and spiropyran moieties to realize the time‐dependent encryption of information based on the density of the host–guest crosslinks.^[^ ^19^ ^]^ Considering the above studies, although great progress has been made in the fabrication of such systems in the solid state, the development of a photoreversible RTP–fluorescence switch with tunable long‐lived multicolor emission in the aqueous phase has yet to be reported to the best of our knowledge.

**Scheme 1:**
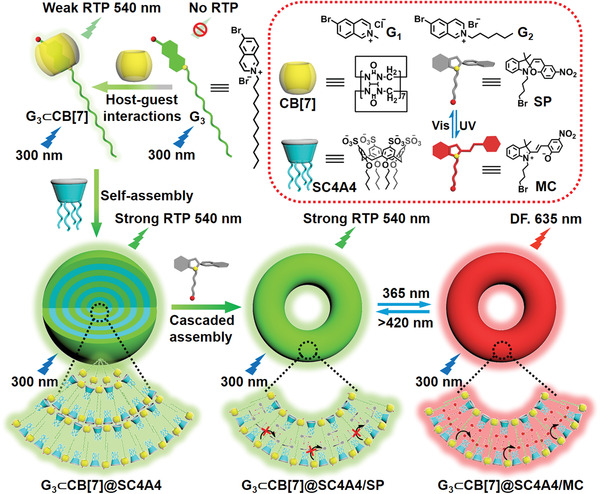
Schematic illustration of the construction of a highly reversible phosphorescence/fluorescence photoswitch based on a cascaded supramolecular assembly process in aqueous solution.

Thus, we herein report the construction of a cascaded supramolecular multivalent assembly composed of two macrocyclic molecules, namely cucurbit[7]uril (CB[7]) and sulfonatocalix[4]arene (SC4A4), in addition to a phosphor 6‐bromoisoquinoline derivative (G_3_), and a photoswitchable spiropyran (SP) moiety, with the aim of achieving reversible photoswitching between RTP and delayed fluorescence by virtue of a light‐driven supramolecular RTP energy transfer in the aqueous phase (**Scheme** **1**). To demonstrate the potential applicability of this photoreversible multivalent assembly exhibiting RTP–fluorescence switching, the multicolor labeling of A549 cancer cells is attempted.

## 2 Results and Discussion

**Figure 1:**
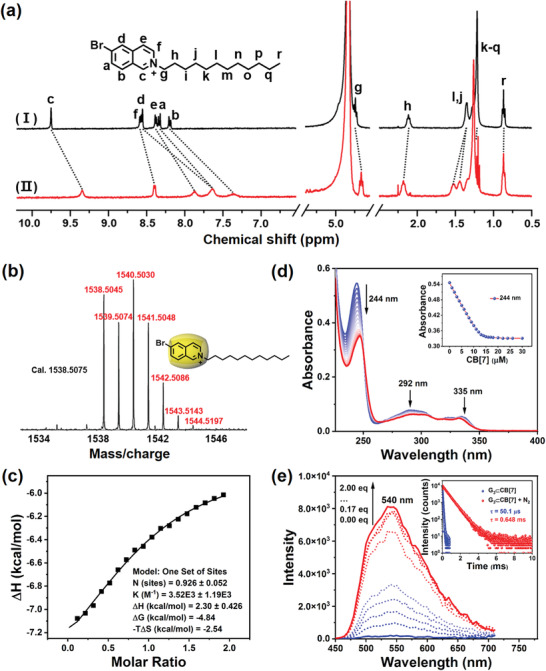
a) Partial ^1^H NMR spectra (400 MHz, D_2_O, 298 K) of (I) free G_3_ ([G_3_] = 1.0 × 10^−3^ m) and (II) G_3_⊂CB[7] ([G_3_] = 1.0 × 10^−3^ m, [CB[7]] = 1.5 × 10^−3^ m). b) High‐resolution MALDI‐TOF mass spectrum of G_3_⊂CB[7]. The peak at *m/z* 1538.5045 correspond to [M−Br^−^]^+^. *m/z* calcd for C_63_H_73_N_29_O_14_Br^+^ 1538.5075. Inset: The possible host–guest binding mode of G_3_⊂CB[7]. c) ITC isotherms were obtained for the titration of G_3_ with CB[7] in water at 298 K. d) UV–vis absorption spectra and (inset) absorbance intensity changes observed for G_3_ at 244 nm in water at 298 K upon the addition of CB[7] ([G_3_] = 1.0 × 10^−5^ m and [CB [7]] = 0–3.0 × 10^−5^ m). e) Phosphorescence emission spectral changes (delay = 50 µs) for G_3_ at 540 nm in water at 298 K upon the addition of 0, 0.17, 0.33, 0.50, 0.67, 1.0, 1.33, 1.67, and 2.0 equivalents of CB[7] ([G_3_] = 5.0 × 10^−5^ m, *λ*_ex_ = 300 nm). Inset: Time‐resolved photoluminescence decay spectra of G_3_⊂CB[7] at 540 nm in H_2_O at 298 K under air and under a N_2_ atmosphere.

The first challenge in the development of such systems is the discovery of suitable organic phosphorescent molecules for the preparation of an effective multivalent assembly. Inspired by the pioneering work of Tian and coworkers,^[^ ^20^ ^]^ we investigated the excellent optical properties of a bromo‐substituted isoquinoline. More specifically, 6‐bromoisoquinoline derivatives (G_1_–G_3_, Scheme 1) were selected as phosphorescent guest molecules wherein the N‐termini were modified with alkyl chains of different lengths to yield diverse amphiphilic properties. The effects of the aliphatic chain length on the further assembly of G_1_–G_3_ with two macrocyclic molecules (i.e., CB[7] and SC4A4) were then examined, as were the resulting phosphorescence behaviors of the assemblies in aqueous solution. The synthetic routes toward G_1_–G_3_ are presented in Schemes S1–S3 (Supporting Information), and their characterization by ^1^H NMR spectroscopy, ^13^C NMR spectroscopy, and high‐resolution mass spectrometry (HR‐MS) are given in Figures S1–S7 in the Supporting Information. Previously, it was reported that CB[7], possessing a favorable water solubility and a rigid cavity, can form a stable inclusion complex with many positively‐charged guest molecules through cation–dipole and hydrophobic interactions.^[^ ^21^ ^]^ The host–guest complexation behaviors between G_1_–G_3_ and CB[7] were preliminarily investigated by HR‐MS, wherein an intense peak centered on [M−Br^−^]^+^ (*m/z*: 1384.3377, 1454.4127, and 1538.5075) was clearly detected for G_1_⊂CB[7], G_2_⊂CB[7], and G_3_⊂CB[7], which corresponded with the calculated values of 1384.3354, 1454.4136, and 1538.5045, thereby confirming the 1:1 host–guest complexation (**Figure** **1**b; Figures S10 and S11, Supporting Information). To further examine the host–guest binding mode, ^1^H NMR titration experiments were conducted. As shown in Figure 1a and Figure S12 (Supporting Information), upon the gradual addition of CB[7] to G_3_, all aromatic protons of the isoquinoline moiety (i.e., H_a_–H_f_) and H_g_ exhibited an apparent upfield shift because of the shielding effect attributed to its deep encapsulation in the cavity of CB[7]. In contrast, the aliphatic protons (i.e., H_h_–H_r_) exhibited a downfield shift due to the deshielding effect. These shift resemble those observed upon complexation between G_1_ and CB[7], with the exception that the methyl protons of G_1_ exhibited a slight downfield shift (Figure S13, Supporting Information). However, unique differences were observed for the shifts of the G_2_ proton signals, wherein the protons assigned to the aliphatic chain underwent obvious upfield shifts, while the signals corresponding to the aromatic isoquinoline protons remained relatively constant (Figure S14, Supporting Information). These observations indicate that the hexyl substituent docked to CB[7] through hydrophobic interactions.^[^ ^22^ ^]^ In addition, bare G_1_ and G_3_ were found to exhibit three main UV–vis absorption peaks centered at 244, 293, and 335 nm, whose absorbance intensities decreased linearly upon the stepwise addition of CB[7] up to 1.5 equivalents, prior to levelling out (Figure 1d; Figure S15, Supporting Information). In contrast, the main absorbance peak of G_2_ at 244 nm increased with the stepwise addition of CB[7] up to 1.3 equivalent prior to subsequently decreasing (Figure S16, Supporting Information). This unusual phenomenon also indicates the different assembly mode between G_2_ and CB[7]. Moreover, a 1:1 stoichiometric ratio between G_3_ and CB[7] was indicated by the Job plot, in which the maximum was observed at a molar fraction of 0.5 (Figure S17, Supporting Information). Isothermal titration calorimetry (ITC) experiments were then performed to evaluate the binding model between G_3_ and CB[7], and the stoichiometry for the G_3_⊂CB[7] complex was confirmed to be 1:1 with an association constant *K*_s_ of 3.52 × 10^3^ m ^−1^; this result is consistent with previous reports (Figure 1c; Figure S18, Supporting Information).^[^ ^20^ ^]^

After verifying the binding modes of G_1_–G_3_ with CB[7], the optical properties of the corresponding host–guest complexes were studied by photoluminescence spectroscopy in aqueous solution. More specifically, a solution of G_3_ showed an apparent emission peak at 390 nm, whereas a new emission peak appeared at 540 nm upon the continuous addition of CB[7] into the G_3_ solution. This peak gradually increased in intensity during the addition of CB[7], and was accompanied by a simultaneous decrease in the emission intensity at 390 nm (Figure S19, Supporting Information). In addition, time‐gated emission spectroscopy (delay time = 50 µs) revealed that the titration of G_3_ with increasing equivalents of CB[7] resulted in a gradual emergence of an intense peak with a maximum at 540 nm, whose intensity reached a plateau at ≈1.33 equivalents of CB[7] (Figure 1e). Only this single emission peak at 540 nm was observed in the gated spectra, which is indicative of a long‐life emission at 540 nm and short‐life emission at 390 nm (Figure S20, Supporting Information). Further analysis of the time‐resolved PL decay data suggested that the lifetimes at 390 nm were on the nanosecond scale for both G_3_ (1.45 ns) and G_3_⊂CB[7] (4.73 ns), revealing that this peak belongs to a typical fluorescence emission (Figures S21 and S22, Supporting Information). However, the lifetime at 540 nm was measured to be 50.1 µs according to the time‐resolved photoluminescence decay curves (Figures S23 and S24a, Supporting Information). It was also found that the emission intensity was markedly enhanced by approximately 26 times and the lifetime could be further extended to 0.648 ms when bubbling nitrogen (N_2_) through the solution, and this was attributed to the suppression of triplet exciton quenching, thereby confirming that this emerging peak was assigned to solution‐phase phosphorescence (Figure 1e, inset, and Figures S24b and S25, Supporting Information). These results suggest that the tight inclusion G_3_ by CB[7] can immobilize the phosphor particles, restrict vibrations, and suppress both intermolecular collision and vibration dissipation, thereby facilitating the generation of phosphorescence. Similarly, the free G_1_ and G_2_ guests failed to exhibit any distinct phosphorescence emission, although an obvious phosphorescent enhancement comparable to that of G_3_ was observed for G_1_ after complexation with CB[7] under the same conditions (Figures S26 and S27, Supporting Information). In contrast, G_2_ exhibited only a slight increase in its phosphorescent intensity upon complexation with CB[7] (Figure S28, Supporting Information), which was ascribed to the inefficient encapsulation of its 6‐bromoisoquinoline moiety by CB[7], as also suggested by the NMR results discussed above.

**Figure 2:**
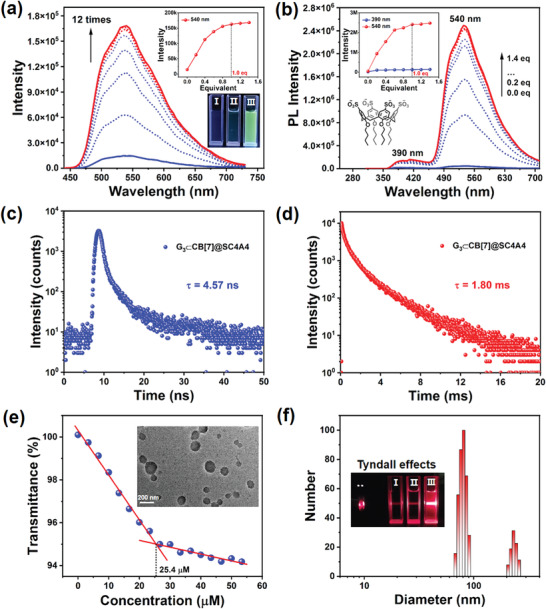
a) Phosphorescence emission spectra (delay = 50 µs) and emission intensity changes observed for G_3_⊂CB [7] at 540 nm in water at 298 K upon the addition of 0, 0.2, 0.4, 0.6, 0.8, 1.0, 1.2, and 1.4 equivalents of SC4A4 ([G_3_] = 5.0 × 10^−5^ m, *λ*_ex_ = 300 nm). Inset: Photographic image of the aqueous solutions of (I) free G_3_, (II) G_3_⊂CB[7], and (III) G_3_⊂CB[7]@SC4A4 upon illumination with UV light under ambient conditions. b) The prompt photoluminescence spectra and photoluminescence emission intensity changes observed for G_3_⊂CB[7] at 390 and 540 nm in water at 298 K upon the addition of 0, 0.2, 0.4, 0.6, 0.8, 1.0, 1.2, and 1.4 equivalents of SC4A4. Inset: Chemical structure of SC4A4. c) Time‐resolved photoluminescence decay spectrum of G_3_⊂CB[7]@SC4A4 at 390 nm in water at 298 K. d) Time‐resolved photoluminescence decay spectrum of G_3_⊂CB[7]@SC4A4 at 540 nm in water at 298 K. e) Dependence of the optical transmittance at 450 nm upon the addition of SC4A4 to an aqueous solution of G_3_⊂CB[7] at 298 K. Inset: Transmission electron microscopy image of the G_3_⊂CB [7]@SC4A4 assembly. f) Dynamic light scattering analysis of the G_3_⊂CB[7]@SC4A4 assembly. Inset: Tyndall effects of (I) free G_3_, (II) G_3_⊂CB[7], and (III) G_3_⊂CB[7]@SC4A4.

It has been reported that the inherently amphipathic sulfonatocalix[4]arenes can be employed to form multivalent supramolecular assemblies to produce a pronounced enhancement in both the fluorescent and phosphorescent emissions of organoluminescent molecules in the aqueous phase.^[^ ^12^, ^23^ ^]^ Thus, after elucidating the complexation‐induced phosphorescence emission behaviors of the complexes formed between G_1_–G_3_ and CB[7], the amphipathic sulfonatocalix[4]arene was introduced to promote a secondary assembly with G1–G3⊂CB[7] with the intention of improving their original phosphorescent performances. For this purpose, three sulfonatocalix[4]arene derivatives, including lower‐rim hydroxyl‐/butyl‐/dodecyl‐modified sulfonatocalix[4]arenes (i.e., SC4A0, SC4A4, and SC4A12), were selected to explore the coassembly processes with G_1_–G_3_⊂CB[7]. Considering that G_3_ contains a relatively long hydrophobic chain with an enhanced water‐repelling segment, we initially studied the assembly behaviors of G_3_⊂CB[7] with these three sulfonatocalix[4]arenes. More specifically, the stepwise addition of SC4A4 to an aqueous solution of G_3_⊂CB[7] led to a gradual enhancement of the phosphorescence intensity at 540 nm, and a plateau was almost reached upon increasing the amount of SC4A4 to 1.0 equivalent. This led to a clear enhancement (≈12 times) in the RTP intensity that could be clearly observed with the naked eye under UV light irradiation (**Figure** **2**a). Similar results were observed for the photoluminescence spectra, with the exception that the fluorescence intensity remained unchanged at 390 nm with an average lifetime of 4.57 ns (Figure 2b,c). Moreover, the RTP lifetime at 540 nm was found to be dramatically enhanced (≈36 times) from 50.1 µs to 1.80 ms, and a further increase to 3.00 ms was observed when N_2_ was bubbled into the aqueous solution (Figure 2d; Figures S29 and S30, Supporting Information). In addition, the phosphorescence quantum yield of G_3_⊂CB[7] increased from 1.01% to 20.2% following its coassembly with SC4A4 (Figure S31, Supporting Information). In comparison, the direct assembly of SC4A4 with G_3_ resulted in negligible phosphorescence, thereby demonstrating that the complexation of G_3_ with CB[7] plays an indispensable role in inducing its phosphorescence (Figure S32, Supporting Information). Importantly, these results demonstrate that the addition of SC4A4 enhanced the phosphorescence emission as opposed to disrupting the G_3_⊂CB[7] complex. These effects can be mainly attributed to the hydrophobic environment created after coassembly, which not only provided a protective environment for the phosphors (i.e., to avoid attacks from water or dissolved oxygen species), but also further suppressed nonradiative relaxation decay.

In control experiments, it was found that the secondary assemblies with SC4A4 failed to significantly increase the original phosphorescence emission for both G_1_⊂CB[7] and G_2_⊂CB[7], and this was particularly true in the case of G_2_⊂CB[7] (Figures S33–S36, Supporting Information). These results further confirm that the effective encapsulation by CB[7] and the intrinsic amphipathic nature of the phosphor contribute to the coassembly process with SC4A4 to yield a highly efficient phosphorescence emission. The length of the aliphatic chains on the lower‐rim of sulfonatocalix[4]arene is also key when considering the interaction of them with G_3_⊂CB[7]. Although both SC4A0 and SC4A12 strengthened the phosphorescence emission of G_3_⊂CB[7], the ultimate emission intensity was lower than that achieved using SC4A4 (Figures S37 and S38, Supporting Information). These differences could be readily distinguished from photographic images of their aqueous solutions under UV light irradiation (Figures S39 and S40, Supporting Information). It should be noted here that we also investigated the effects of some common small molecular surfactants, such as sodium dodecyl sulfate and cetyltrimethylammonium bromide, but these compounds were found to have no enhancement effect on the RTP performance of G_3_⊂CB[7] (Figure S41, Supporting Information).

In addition, the assembly process between G_3_⊂CB[7] and SC4A4 was studied by monitoring changes in the optical transmittance. More specifically, based on the transmittance changes at 450 nm and at a fixed concentration of G_3_⊂CB[7], the critical aggregation concentration was determined to be 25.4 μμ for SC4A4 (Figure 2e; Figure S42, Supporting Information). Transmission electron microscopy (TEM), dynamic light scattering (DLS), and zeta potential experiments were then conducted to examine the detailed morphological structures after assembly. For example, TEM images showed that G_3_⊂CB[7] self‐assembled to produce many small irregular nanoaggregates (Figure S43, Supporting Information), while G_3_⊂CB[7]@SC4A4 existed as numerous close‐packed spherical nanoparticles with the diameter approximately 60–250 nm (Figure 2e, inset). In addition, G_3_⊂CB[7]@SC4A4 displayed the most obvious Tyndall effect when compared with free G_3_ and G_3_⊂CB[7], further confirming the formation of large assemblies (Figure 2f, inset). Furthermore, DLS experiments revealed that the majority of obtained nanoparticles were ≈80 nm in diameter and a few nanoparticles with diameter of ≈230 nm also existed, which was consistent with the TEM results (Figure 2f). In contrast with G_3_⊂CB[7], whose zeta potential was measured as 30.8 mV, the zeta potential of G_3_⊂CB[7]@SC4A4 was −23.3 mV, thereby indicating the presence of negatively charged surfaces on the latter, which could ensure the stability and dispersibility of such aggregates in biological imaging (Figure S44, Supporting Information). These results further confirm that G_3_⊂CB[7] and SC4A4 could indeed co‐assemble to form ternary supramolecular nanoparticles courtesy of synergistic noncovalent interactions such as hydrophobic and electrostatic interactions.

**Figure 3:**
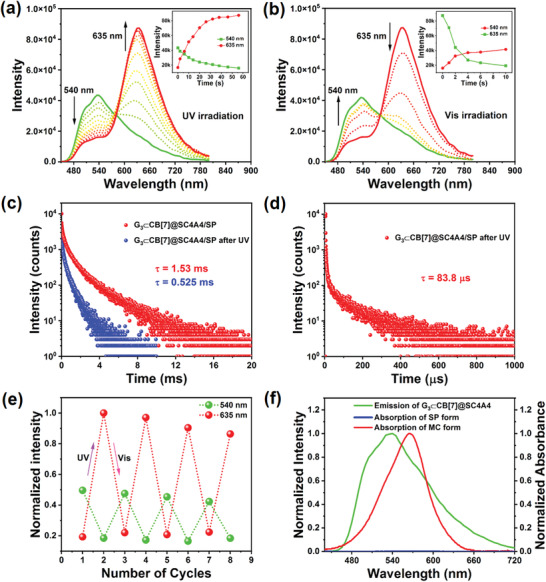
a) Phosphorescence emission spectra (delay = 50 µs) and emission intensity changes for G_3_⊂CB[7]@SC4A4/SP at 540 and 635 nm upon irradiation with UV light (365 nm, 56 s) in water at 298 K. b) Phosphorescence emission spectra (delay = 50 µs) and emission intensity changes for G_3_⊂CB[7]@SC4A4/MC at 540 and 635 nm upon irradiation with visible light (>420 nm, 10 s) in water at 298 K. c) Time‐resolved photoluminescence decay spectra of G_3_⊂CB[7]@SC4A4/SP at 540 nm before and after irradiation with UV light in water at 298 K. d) Time‐resolved photoluminescence decay spectrum of G_3_⊂CB[7]@SC4A4/SP at 635 nm after irradiation with UV light in water at 298 K. e) Phosphorescence emission intensity (delay = 50 µs) changes for G_3_⊂CB[7]@SC4A4/SP at 540 and 635 nm upon alternating irradiation with UV (365 nm, 56 s) and visible light (>420 nm, 10 s) in water at 298 K ([G_3_] = [SC4A4] = [SP] = 5.0 × 10^−5^ m, [CB[7]] = 7.5 × 10^−5^ m, *λ*_ex_ = 300 nm). f) Normalized phosphorescence emission spectrum (left axis) of the G_3_⊂CB[7]@SC4A4 assembly and the partial absorption spectra (right axis) of SP and MC.

Considering the prominently enhanced phosphorescent properties of the G_3_⊂CB[7]@SC4A4 aggregates and their well‐defined stacking nanostructure containing an inner hydrophobic environment, we expected that appropriate hydrophobic fluorescent dyes could function as acceptors and undergo loading into the interiors of these supramolecular nanoparticles. This, in turn, could shorten the distance between donors and acceptors to generate an RTP energy transfer system in aqueous solution. In the present case, the SP unit was added to the phosphorescent system as a photochromic dye to explore its capacity as a receptor by virtue of its light‐controlled reversible conversion between non‐fluorescent SP and fluorescent merocyanine (MC) forms.^[^ ^24^ ^]^ Initially, the possibility of phosphorescence energy transfer from G_3_⊂CB[7]@SC4A4 to the closed‐ring SP form and the open‐ring MC form was probed (**Figure** **3**). Upon exposure to UV light, an absorption peak gradually appeared at 565 nm, which was indicative of the photoisomerization process from the SP to the MC form. This was also clearly distinguished by the color variance of the SP solution from colorless to purple after UV illumination (Figure S45a, Supporting Information). In addition, irradiation with visible light (>420 nm) return the UV absorption properties of the system to their original state, and it was found that this reversible conversion exhibited a good fatigue resistance without any distinctive variations in attenuation under alternating UV and visible light irradiation (Figure S45b–d, Supporting Information). As shown in Figure 3f, the absorption spectrum of the SP form displayed almost no absorption between 450 and 700 nm; however, a clear spectral overlap appeared when the SP unit was transformed into its isomeric MC state, thereby implying that the MC form was an ideal acceptor candidate for phosphorescence energy transfer.

Subsequently, the final quaternary multivalent supramolecular system G_3_⊂CB[7]@SC4A4/SP could be readily acquired through a cascaded assembly process and the encapsulation of SP into the interior hydrophobic region of G_3_⊂CB[7]@SC4A4. TEM and SEM images revealed that after incorporation of the SP derivative, the G_3_⊂CB[7]@SC4A4 supramolecular nanoparticles were converted into donut‐like structures, and DLS data displayed that the assemblies with diameter ≈100 nm existed as the majority component while a few assemblies ≈350 nm in diameter were also found (Figures S46 and S47, Supporting Information). It was observed that upon complexation, the original phosphorescence intensity of G_3_⊂CB[7]@SC4A4 decreased by 8% with a phosphorescence quantum yield of 17.2%, and the phosphorescence lifetime was slightly reduced to 1.53 ms (Figure 3c; Figures S48 and S49a, Supporting Information), which was likely due to a change in the topological morphology of the assembly. Next, the light‐driven RTP energy‐transfer process within G_3_⊂CB[7]@SC4A4/SP was investigated. As indicated in Figure 3a, the phosphorescence intensity at 540 nm gradually decreased upon irradiation with UV light (365 nm, 56 s), and was accompanied by a new emission peak at 635 nm (Figure 3a). The intensity of this new peak remained relatively constant, and it became the dominant emissive peak with a phosphorescence quantum yield of 23.8% (Figure S49b, Supporting Information) when the photostationary state was reached (Figure 3a, inset). This peak corresponded to long‐lived emission because each data point was collected with a delay time of 50 µs to guarantee that the short‐lived fluorescence emission was completely eliminated in the gated spectra. Additionally, after exposure to UV light irradiation, negligible delayed emission was observed at 635 nm for G_3_⊂CB[7]@SC4A4/MC when excited with the optimal wavelength of MC at 550 nm, while excitation at 300 nm gave a remarkable delayed emission at 635 nm under identical experimental conditions (Figure S50, Supporting Information). In a further control experiment, it was found that the G_3_‐free assembly with SP (i.e., CB[7]@SC4A4/SP) after UV light irradiation exhibited almost no delayed fluorescence emission even under selective excitation with the optimal wavelengths, thereby indicating that the gradual occurrence of valid phosphorescence energy transfer in G_3_⊂CB[7]@SC4A4/SP occurred upon stepwise irradiation with UV light (Figure S51, Supporting Information). Moreover, the donut‐like structure of the assembly was maintained after UV light irradiation, as depicted in Figure S52 in the Supporting Information. Furthermore, the phosphorescence intensity remained above 92% after irradiation of the solution with UV light for 90 min, thereby confirming the bleaching resistance and photostability of G_3_⊂CB[7]@SC4A4 (Figure S53, Supporting Information). These results indicate that continuous UV irradiation resulted in a configurational conversion from the closed‐ring SP form to the open‐ring MC form, whose absorption overlapped efficiently with the phosphorescence emission to achieve gradual RTP energy transfer from the triplet state of the phosphors to the MC generated within the nanoparticles.

**Figure 4:**
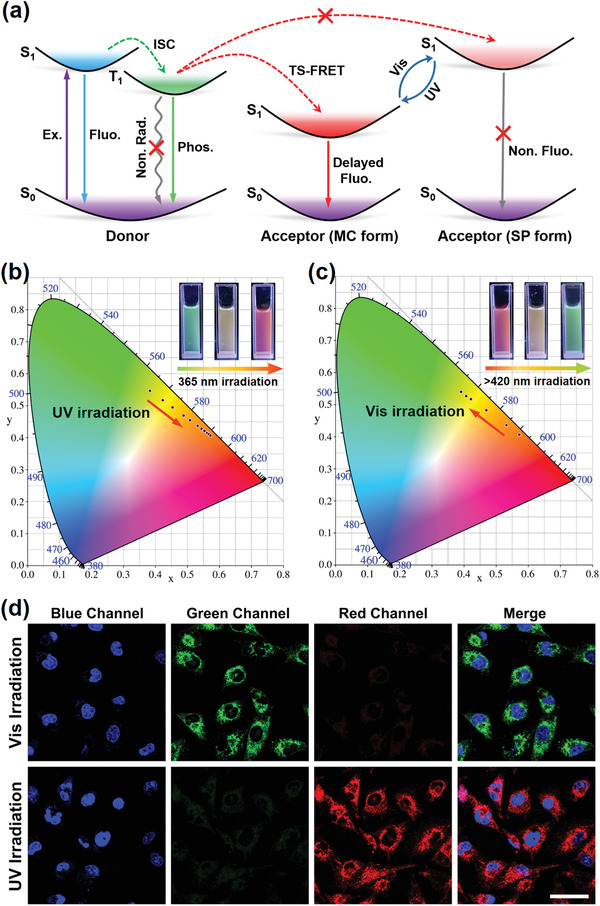
a) Possible working mechanism for the light‐driven supramolecular RTP energy transfer process (Ex. = excitation, Fluo. = fluorescence, Non. Rad. = nonradiation, ISC = intersystem crossing, Phos. = phosphorescence, TS‐FRET = triplet to singlet Förster resonance energy transfer). b) The 1931 CIE chromaticity diagram depicting the color changes and (inset) photographic images of the G_3_⊂CB[7]@SC4A4/SP assembly under continuous UV irradiation, corresponding to Figure 3(a). c) The 1931 CIE chromaticity diagram depicting the color changes and (inset) photographic images of the G_3_⊂CB[7]@SC4A4/MC assembly under continuous visible light irradiation, corresponding to Figure 3(b). d) Cellular imaging of A549 cancer cells costained with the G_3_⊂CB[7]@SC4A4/SP or G_3_⊂CB[7]@SC4A4/MC assembly. 4, 6‐Diamidino‐2‐phenylindole (DAPI, blue) was used to stain the nuclei (scale bar = 30 µm).

Additionally, the lifetime of each emission peak was measured to elucidate the mechanism of the phosphorescence energy transfer process. Time‐resolved decay curves revealed that the phosphorescence lifetime at 540 nm drastically reduced to 0.525 ms after irradiation with UV light (Figure 3c), and that the lifetime of the energy acceptor at 635 nm was in the order of microseconds (i.e., 83.8 µs) while the lifetime of free MC was 1.08 ns (Figure 3d; Figures S54–S56, Supporting Information), which confirmed that phosphorescence energy transfer could be attributed to the triplet‐to‐singlet Förster resonance energy transfer mechanism (TS‐FRET), rather than the simple radiative energy transfer mechanism.^[^ ^15^, ^18^ ^]^ Thus, based on the declined donor lifetime data at 540 nm, the energy transfer efficiency was calculated to be 65.7%. Importantly, the RTP intensity essentially returned to its initial level when the aqueous assembly solution was irradiated with visible light (>420 nm) for 10 s, implying the cutoff of the TS‐FRET pathway (Figure 3b). This phenomenon was mainly derived from the reverse photoisomerization of MC to generate the SP state, which possessed a mismatched absorption with the RTP emission. A possible working mechanism for this light‐driven supramolecular RTP energy transfer process is shown in **Figure** **4**a. Crucially, upon alternating UV and visible light irradiation, it was possible to repeat this light‐controlled RTP energy transfer cycle several times without any apparent decline in performance (Figure 3e; Figure S57, Supporting Information). In addition, this light‐driven RTP energy transfer process could be readily visualized with the naked eye, where the color of the aqueous multivalent supramolecular assembly solution gradually changed from bright green to yellow, and finally to red, with prolonged UV light irradiation (Figure 4b, inset). The CIE 1931 chromaticity diagram (Figure 4b) also illustrates the same linear trend of color change, wherein the coordinates shifted from (0.38, 0.55) to (0.57, 0.41). Subsequently, it was found that the color of the solution returned to green upon further irradiation with visible light (>420 nm) (Figure 4c). For comparison, the performance of light‐driven RTP energy transfer was also examined by doping two different molar ratios of SP into the G_3_⊂CB[7]@SC4A4 assembly. In both cases, a certain degree of phosphorescence energy transfer was observed, with the emission peak at 635 nm emerging upon exposure to UV light; however, the energy transfer efficiencies (i.e., 13.1% and 30.3%) were relatively low (Figures S58 and S59, Supporting Information), and the corresponding lifetimes at 635 nm were 52.4 and 69.0 µs, respectively (Figures S60 and S61, Supporting Information).

The effective photoresponsive RTP energy transfer performance observed for G_3_⊂CB[7]@SC4A4/SP encouraged us to investigate its potential application in multicolor cellular imaging. For this purpose, we initially used the cell counting kit‐8 (CCK‐8) assay to assess the cytotoxicity of both G_3_⊂CB[7]@SC4A4/SP and G_3_⊂CB[7]@SC4A4/MC toward human lung adenocarcinoma cells (A549 cells). As shown in Figure S62 (Supporting Information), the viability of the A549 cells was maintained >90% after incubation with both assemblies (up to 0.06 × 10^−3^ m concentration) for 24 h, thereby implying their relatively low toxicity. Subsequently, the photoswitchable multicolor cellular imaging capability of G_3_⊂CB[7]@SC4A4/SP was investigated using living A549 cells. It was found that the cells treated with G_3_⊂CB[7]@SC4A4/SP emitted a bright green emission signal, while the red channel presented a negligible emission signal after visible light irradiation. These observations were attributed to the fact that the SP moieties in the nanoparticles existed in the nonfluorescent state, and thus were unable to quench the phosphorescence to promote the TS‐FRET process. However, only a faint green signal was observed in the cell cytoplasm, and this was accompanied by a clearly enhanced red emission signal after irradiation with UV light due to transformation of the SP moiety into its fluorescent MC state. As a result, the TS‐FRET process was promoted, and the red signal corresponding to the MC state appeared. These results demonstrate that the obtained quaternary supramolecular nanoparticles can serve as photoswitchable cellular imaging reagents to realize photocontrolled multicolor cell labeling with a low toxicity, therefore highlighting their potential for use as smart labels for biological studies.

## 3 Conclusion

In conclusion, an aqueous‐phase supramolecular RTP–fluorescence switch based on light‐driven phosphorescence energy transfer was successfully constructed via a cascaded assembly strategy in the presence of cucurbit[7]uril (CB[7]) and sulfonatocalix[4]arene (SC4A4), which exhibited phototunable long‐lived multicolor conversion from phosphorescence to delayed fluorescence. Due to its relatively long aliphatic chain, 6‐bromoisoquinoline derivative G_3_ was effectively encapsulated into the rigid cavity of CB[7] to induce the emergence of green phosphorescence emission at 540 nm. Subsequent coassembly with the amphipathic SC4A4 generated compact nanoparticles that exhibited a significant enhancement in the RTP emission behavior (i.e., by ≈12 times) and an extended lifetime of 1.80 ms (c.f., 50.1 µs). Compared with G_3_⊂CB[7], which possessed a phosphorescence quantum yield of 1.01%, G_3_⊂CB[7]@SC4A4 displayed a markedly increased phosphorescence quantum yield of 20.2%. Remarkably, we also found that an spiropyran (SP) unit could be introduced as a photochromic dye to give G_3_⊂CB[7]@SC4A4/SP, which exhibited light‐driven RTP energy transfer under illumination with UV light via the triplet‐to‐singlet Förster resonance energy transfer mechanism process by virtue of the good spectral overlap between RTP emission and its fluorescent merocyanine state. Ultimately, this endowed the final aggregate with multicolor tunable long‐lived emission, thereby rendering it suitable as a cell labeling agent for photocontrolled multicolor imaging, as demonstrated by some preliminary experiments. Such nanostructures possessing unique light‐controlled photoluminescence behaviors therefore provide a novel approach for the creation of stimuli‐responsive phosphorescent materials, and are expected to have great potential for application in the biological field.

## Conflict of Interest

The authors declare no conflict of interest.

## Supporting information

Supporting Information

## Data Availability

The data that support the findings of this study are available from the corresponding author upon reasonable request.
