# Supplementary material for: A Highly Efficient Phosphorescence/Fluorescence Supramolecular Switch Based on a Bromoisoquinoline Cascaded Assembly in Aqueous Solution
Source: Adv Sci (Weinh). 2022 Mar 13;9(14):2200524. doi: 10.1002/advs.202200524 (PMC9108601; doi:10.1002/advs.202200524)
Supplement: Supplementary file 1 — Supporting Information [file ADVS-9-2200524-s001.pdf]

ADVANCED SCIENCE
Open Access

Supporting Information

for *Adv. Sci.*, DOI 10.1002/advs.202200524

A Highly Efficient Phosphorescence/Fluorescence Supramolecular Switch Based on a Bromoisoquinoline Cascaded Assembly in Aqueous Solution

*Xian-Yin Dai, Yu-Yang Hu, Yonghui Sun, Man Huo, Xiaoyun Dong and Yu Liu**

Supporting Information

A Highly Efficient Phosphorescence/Fluorescence Supramolecular Switch Based on a Bromoisoquinoline Cascaded Assembly in Aqueous Solution

*Xian-Yin Dai, Yu-Yang Hu, Yonghui Sun, Man Huo, Xiaoyun Dong and Yu Liu**

Dr. X.-Y. Dai, Dr. Y.-Y. Hu, Dr. Y. Sun, Dr. M. Huo, Dr. X. Dong, Prof. Y. Liu

College of Chemistry, State Key Laboratory of Elemento-Organic Chemistry, Nankai University, Tianjin 300071, P. R. China

E-mail: yuliu@nankai.edu.cn

Table of Contents

1. Measurements ……S2

2. Synthetic Protocols ……S3

3. Investigation of host-guest properties between G_1/G_2 and CB[7] ……S11

4. Studies on optical behaviors of G_1-G_3 after complexation with CB[7] and cascaded assembly with SC4A4 ……S17

5. Characterizations of G_3⊂CB[7]@SC4A4 and G_3⊂CB[7]@SC4A4/SP assembly ……S25

6. Phosphorescence energy transfer measurements of G_3⊂CB[7]@SC4A4/SP ……S29

7. Reference ……S34

1. Measurements

All chemicals were obtained from commercial suppliers unless noted otherwise. ^{1}H NMR (400 MHz) and ^{13}C NMR (100 MHz) were carried out on a Bruker Avance spectrometers. The mass spectra of the compounds were recorded on Varian 7.0T FTMS with the MALDI ion source. UV–vis spectra and optical transmittance were recorded in a quartz cell (light path: 10 mm) on a Shimadzu UV-3600 spectrophotometer equipped with a PTC-348WI temperature controller. Photoluminescence spectra and lifetimes were obtained on FLS980 instrument (Edinburg Instruments, Livingstone, UK). The phosphorescent efficiency is measured by using microsecond lamp (delay 50 μs) in order to filter the interference of fluorescence. The sample solution (3 mL) was filtered through a 0.45 μm Millipore filter and then examined on a laser light scattering spectrometer (BI-200SM) equipped with a digital correlator (Turbo Corr) at 636 nm at a scattering angle of 90°. The hydrodynamic diameter (Dh) was determined by DLS experiments at 25°C. Zeta potential analysis was performed on a Brookhaven ZetaPALS (Brookhaven Instrument, USA) at 298 K in H_2O. The TEM images were recorded by a high-resolution transmission electron microscope (Philips Tecnai G2 20S-TWIN microscope) operating at an accelerating voltage of 200 keV. The sample for high-resolution TEM measurements was prepared by dropping the solution onto a copper grid. The grid was then air-dried. The SEM images were recorded on a JEOL JSM-7500F scanning electronic microscope operating at an accelerating voltage of 30 keV. CCK8 assay were detected by Microplate Reader (American BioTek Synergy 4). Microsoft 2013 and OriginPro 2020b were used for data analysis.

Cell Culture: The human lung cancer cell line A549 cells were all purchased from Cell Resource Center, Chinese Academy of Medical Science Beijing. A549 cancer cells were incubated by using Ham's F12 nutrient medium supplemented with 10 % FBS and 1 % penicillin/streptomycin in a humidified incubator with 5% CO_2 atmosphere at 37°C. Before being used in experiments, all cells were pre-cultured to achieve confluence.

Cellular imaging: A549 cells were first subcultured into confocal petri dish and incubated for 24 h at 37 °C in a humidified 5% CO_2 atmosphere, then G_3⊂CB[7]@SC4A4/SP or G_3⊂CB[7]@SC4A4/MC were added into the dish to ensure their concentration at 30 μM in

culture medium and cultured for another 24 h. After that, the culture medium was discarded and the cells were washed with 0.01 M PBS at least three times. Finally, the cells were subjected to observation by fluorescence microscope. The luminescence was shown as green channel collected from 500-550 nm and red channel collected from 600-650 nm.

Cytotoxicity experiments. First, A549 cells were plated at 96-well plates and incubated for 24 h. After that, the cells were treatment with G_3⊂CB[7]@SC4A4/SP or G_3⊂CB[7]@SC4A4/MC at a serial of concentrations from 0 to 60 μM for another 24 h in the absence of light. Subsequently, the culture medium was discarded and the cells were washed with 0.01 M PBS twice. Then CCK8 solution was added to each well and the cells were further incubated at 37 °C for 1 h. The absorbance value was obtained by a microplate reader at 450 nm wavelength. The data were all displayed as the mean ± standard deviation.

Statistical Analysis. Three replicates of each experiment were performed, and values herein stand for means ± standard deviations (SD). Differences between groups were compared by a one-way analysis of variance test ($p < 0.05$). All statistical tests were performed using the SPSS software package (ver. 20, IBM, USA).

2. Synthetic Protocols

SC4A4 was synthesized according to published literature.[S1] ^{1}H NMR (400 MHz, D_2O, ppm) δ 7.22 (s, 2H), 4.48 (d, J = 13.2 Hz, 1H), 3.96 (t, J = 7.5 Hz, 2H), 3.33 (d, J = 13.2 Hz, 1H), 1.89 (dt, J = 15.2, 7.5 Hz, 2H), 1.40 (dq, J = 14.5, 7.2 Hz, 2H), 0.91 (t, J = 7.4 Hz, 3H).

SP was synthesized according to published literature.[S2] ^{1}H NMR (400 MHz, DMSO-d_6, 298 K) δ 8.22 (s, 1H), 8.00 (d, J = 8.9 Hz, 1H), 7.22 (d, J = 10.2 Hz, 1H), 7.11 (s, 2H), 6.86 (d, J = 8.9 Hz, 1H), 6.79 (t, J = 7.3 Hz, 1H), 6.63 (d, J = 8.0 Hz, 1H), 6.00 (d, J = 10.3 Hz, 1H), 3.51 (t, J = 5.7 Hz, 2H), 3.21-3.03 (m, 2H), 1.85-1.72 (m, 2H), 1.62 (s, 2H), 1.20 (s, 3H), 1.10 (s, 3H).

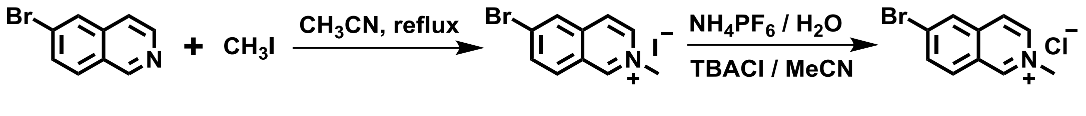

Scheme S1. Synthetic route of compound G_1.

Synthesis of compound G_1: 6-Bromoisoquinoline (1.0 mmol, 208 mg, 1.0 eq) and

iodomethane (3.0 mmol, 425.6 mg, 3.0 eq) were dissolved in anhydrous CH_3CN (20 mL). And the reaction mixture was then heated to reflux overnight under N_2 atmosphere. Rotary evaporation was used to remove the solvents, and white precipitates were obtained when a large amount of diethyl ether was added and then collected by filtration. The obtained white precipitates were re-dissolved in a small amount of water, 326 mg ammonium hexafluorophosphate was added and precipitate was appeared, filtered to get solid. Then the solid was dissolved into 10 mL CH_3CN, 556 mg tetrabutylammonium chloride was added and white precipitate was appeared, filtered to get solid and the product was washed with CH_3CN for 3 times respectively to yield compound **G_1** as white solid (yield: 76%). ^{1}H NMR (400 MHz, DMSO-d_6, ppm) δ 10.24 (s, 1H), 8.82 (dd, J = 6.8, 0.9 Hz, 1H), 8.71 (d, J = 1.3 Hz, 1H), 8.52 (d, J = 6.8 Hz, 1H), 8.44 (d, J = 8.8 Hz, 1H), 8.20 (dd, J = 8.8, 1.8 Hz, 1H), 4.48 (s, 3H); ^{13}C NMR (100 MHz, DMSO-d_6, ppm) δ 151.07 (s), 137.49 (s), 137.04 (s), 134.30 (s), 132.01 (s), 131.21 (s), 129.54 (s), 125.76 (s), 124.33 (s), 47.93 (s).

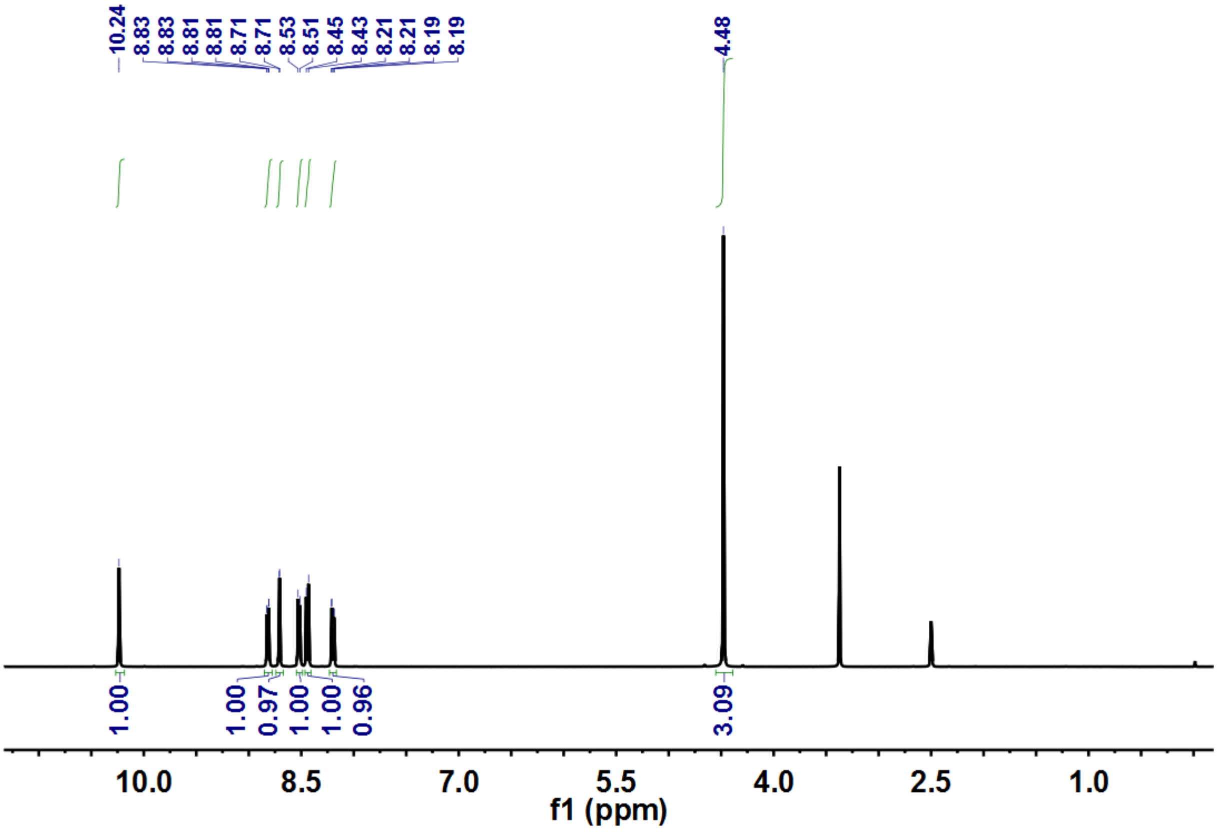

Figure S1. ^{1}H NMR spectrum of compound G_1 (400 MHz, DMSO-d_6, 298 K).

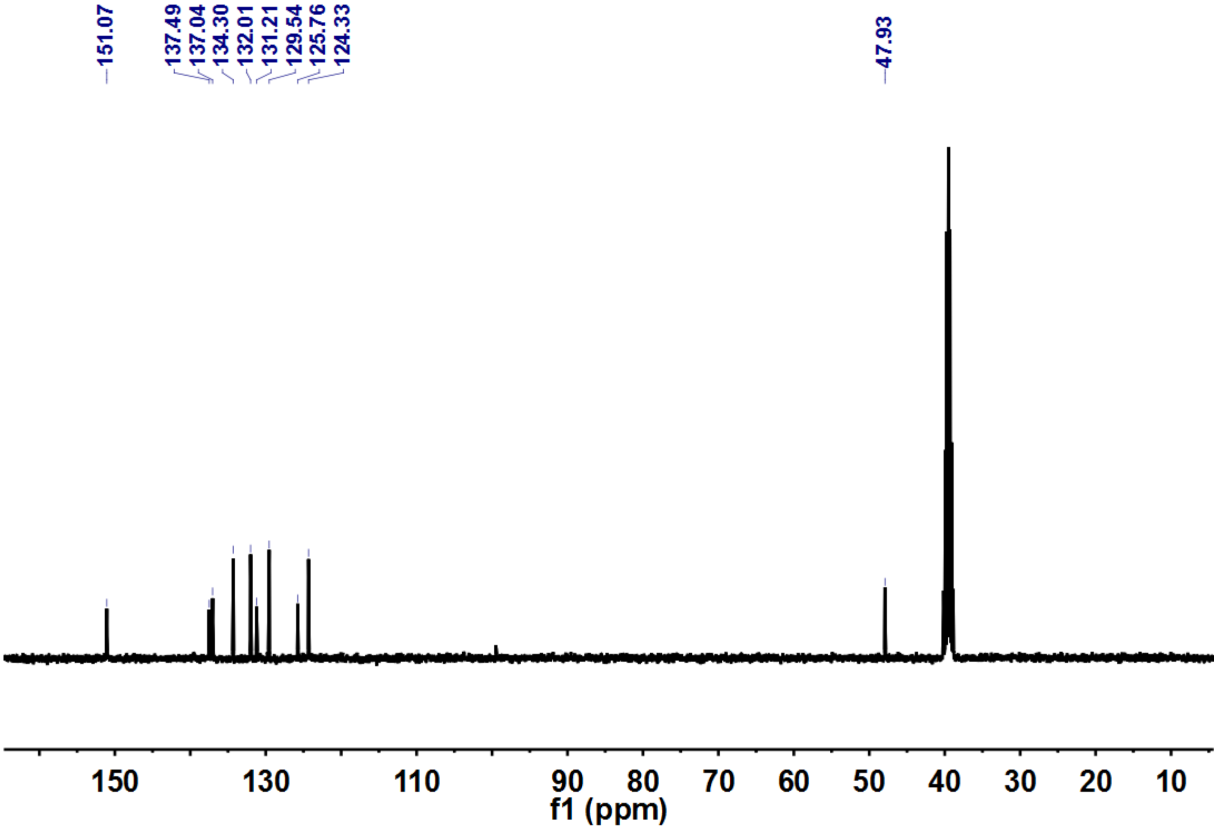

Figure S2. ^{13}C NMR spectrum of compound G_1 (100 MHz, DMSO-d_6, 298 K).

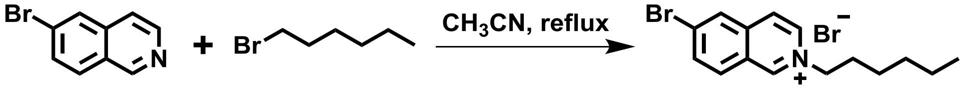

Scheme S2. Synthetic route of compound G_2.

Synthesis of compound G_2: 6-Bromoisoquinoline (1.0 mmol, 208 mg, 1.0 eq) and 1-Bromohexane (3.0 mmol, 495.2 mg, 3.0 eq) were dissolved in anhydrous CH_3CN (20 mL). And the reaction mixture was then heated to reflux overnight under N_2 atmosphere. The excess solvents were removed by rotary evaporation and the residues were washed with a large amount of diethyl ether and then collected by filtration to yield compound G_2 as faint yellow solid (yield: 74%). ^1H NMR (400 MHz, DMSO-d_6, ppm) δ 10.19 (s, 1H), 8.89 (d, J = 6.5 Hz, 1H), 8.71 (s, 1H), 8.54 (d, J = 6.5 Hz, 1H), 8.43 (d, J = 8.7 Hz, 1H), 8.23 (d, J = 8.5 Hz, 1H), 4.71 (t, J = 7.0 Hz, 2H), 2.01 (s, 2H), 1.30 (s, 6H), 0.85 (s, 3H); ^{13}C NMR (100 MHz, DMSO-d_6, ppm) δ 150.21 (s), 137.78 (s), 136.05 (s), 134.39 (s), 132.18 (s), 131.42 (s), 129.57 (s), 125.97 (s), 124.82 (s), 60.83 (s), 30.57 (s), 30.31 (s), 25.10 (s), 21.81 (s), 13.80 (s).

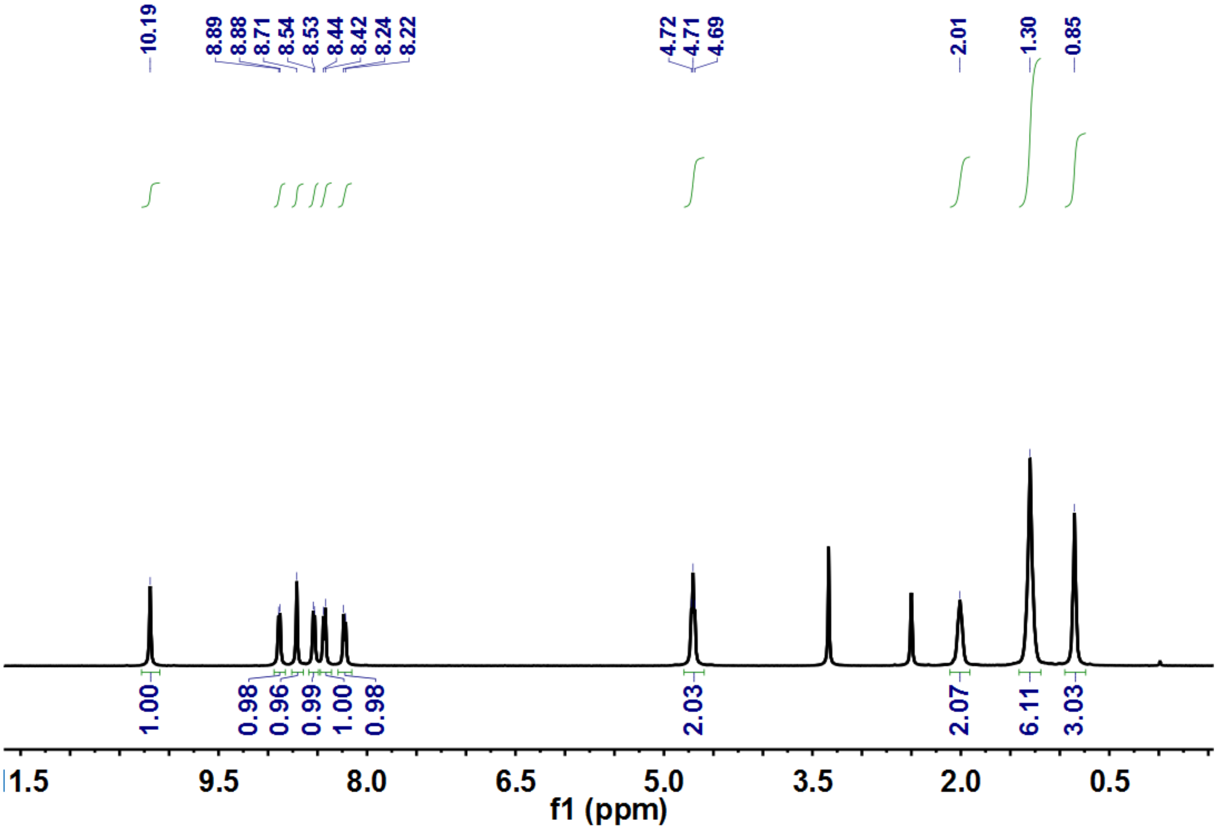

Figure S3. ^{1}H NMR spectrum of compound G_2 (400 MHz, DMSO-d_6, 298 K).

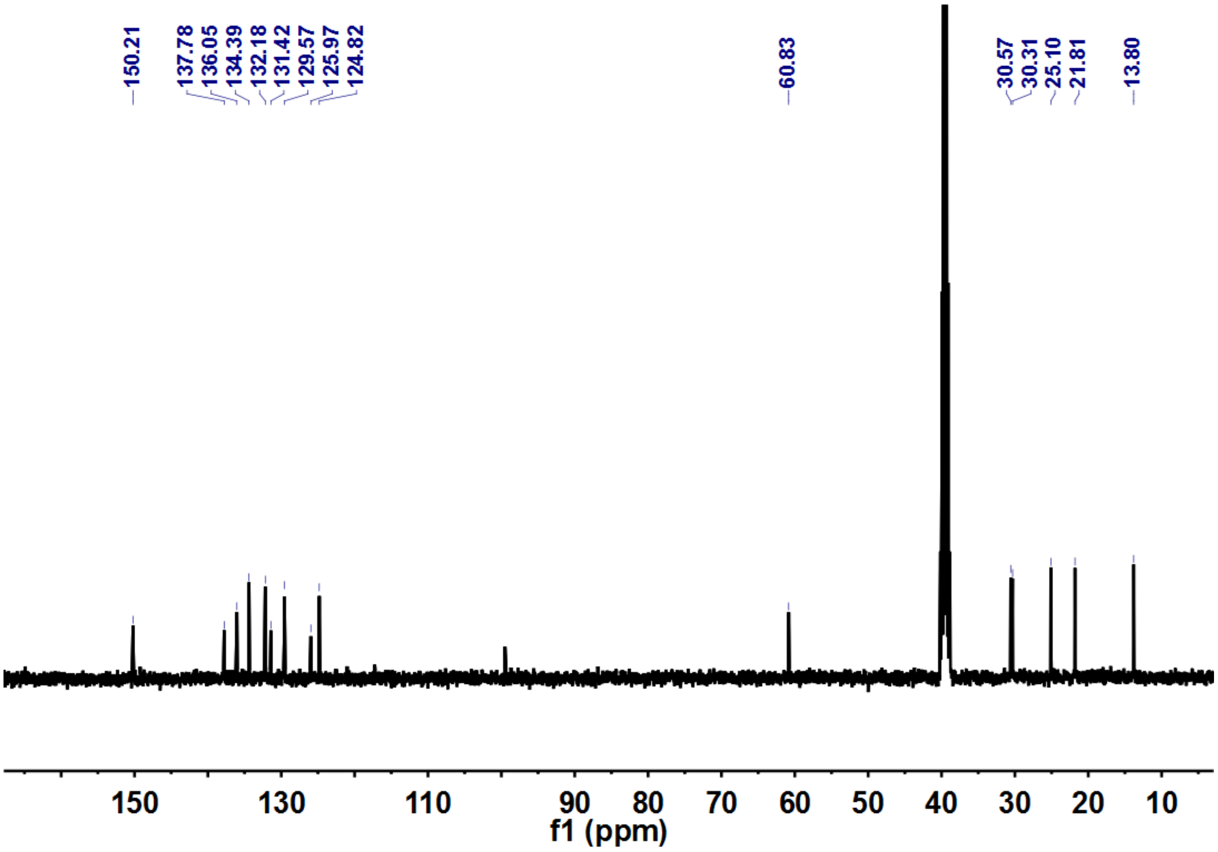

Figure S4. ^{13}C NMR spectrum of compound **G_2** (100 MHz, DMSO-d_6, 298 K).

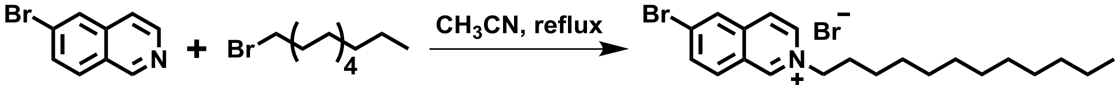

Scheme S3. Synthetic route of compound G_3.

Synthesis of compound G_3: 6-Bromoisoquinoline (1.0 mmol, 208 mg, 1.0 eq) and 1-Bromododecane (3.0 mmol, 747.7 mg, 3.0 eq) were dissolved in anhydrous CH_3CN (20 mL). And the reaction mixture was then heated to reflux overnight under N_2 atmosphere. The excess solvents were removed by rotary evaporation and the residues were washed with a large amount of diethyl ether and then collected by filtration to yield compound G_3 as cream-colored solid (yield: 71%). ^{1}H NMR (400 MHz, DMSO-d_6, ppm) δ 10.09 (s, 1H), 8.84 (d, J = 6.8 Hz, 1H), 8.70 (s, 1H), 8.51 (d, J = 6.8 Hz, 1H), 8.41 (d, J = 8.8 Hz, 1H), 8.23 (dd, J = 8.8, 1.8 Hz, 1H), 4.68 (t, J = 7.4 Hz, 2H), 2.00 (s, 2H), 1.26 (d, J = 35.2 Hz, 18H), 0.85 (t, J = 6.8 Hz, 3H); ^{13}C NMR (100 MHz, DMSO-d_6, ppm) δ 150.21 (s), 137.78 (s), 136.05 (s), 134.40 (s), 132.18 (s), 131.44 (s), 129.57 (s), 125.97 (s), 124.82 (s), 60.86 (s), 31.25 (s), 30.32 (s), 28.95 (s), 28.85 (s), 28.71 (s), 28.66 (s), 28.38 (s), 25.41 (s), 22.05 (s), 13.92 (s); HRMS (ESI): *m/z* calcd for $C_{21}H_{31}BrN^+$ 376.1634 $[M – Br^-]^+$; found: 376.1650.

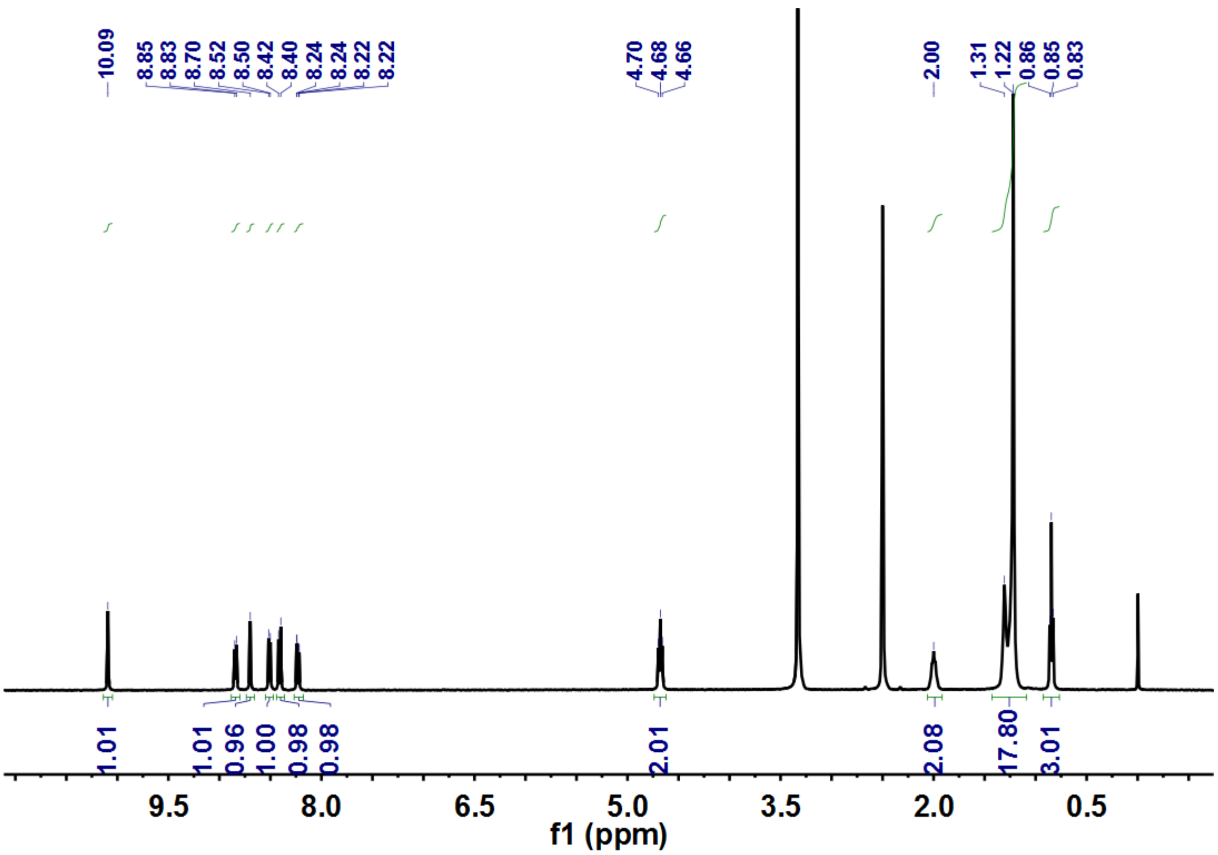

Figure S5. ^{1}H NMR (400 MHz, DMSO-d_6, 298 K) spectrum of compound G_3.

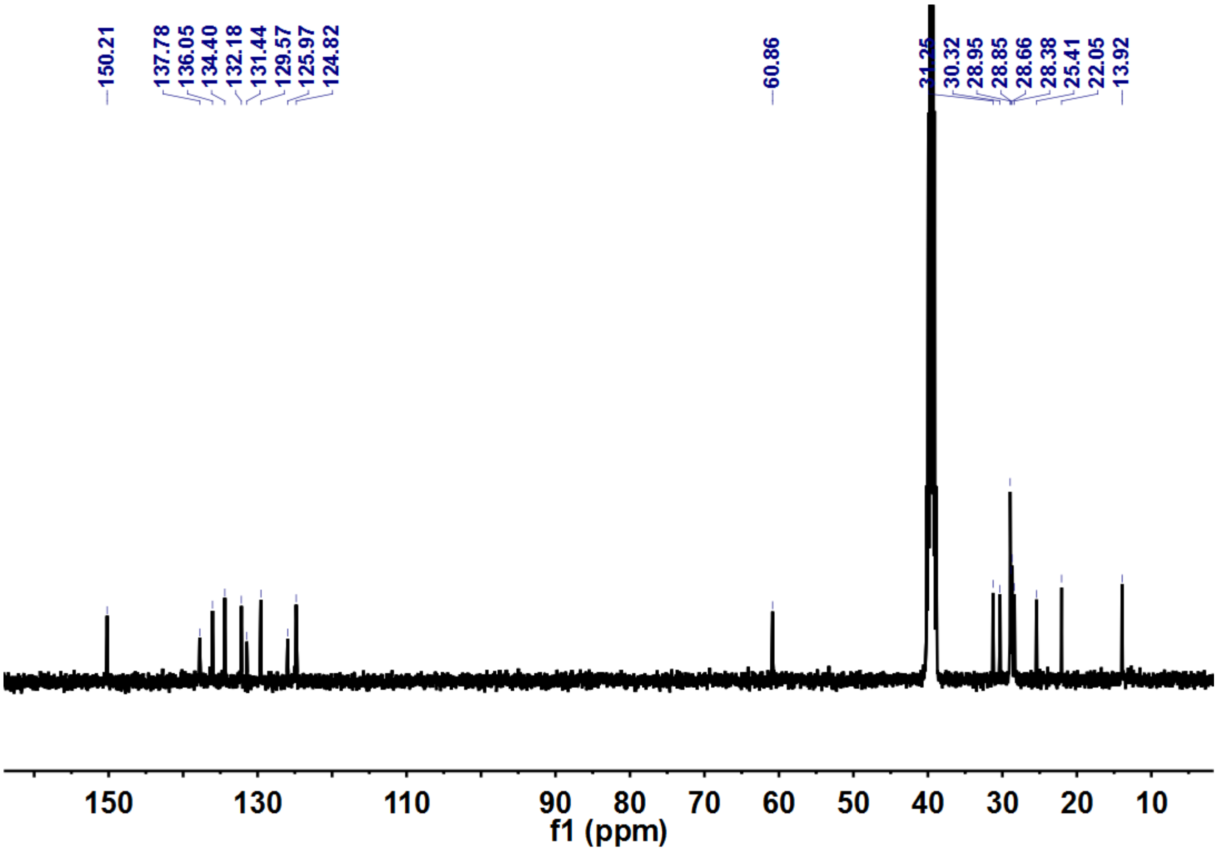

Figure S6. ^{13}C NMR spectrum of compound G_3 (100 MHz, DMSO-d_6, 298 K).

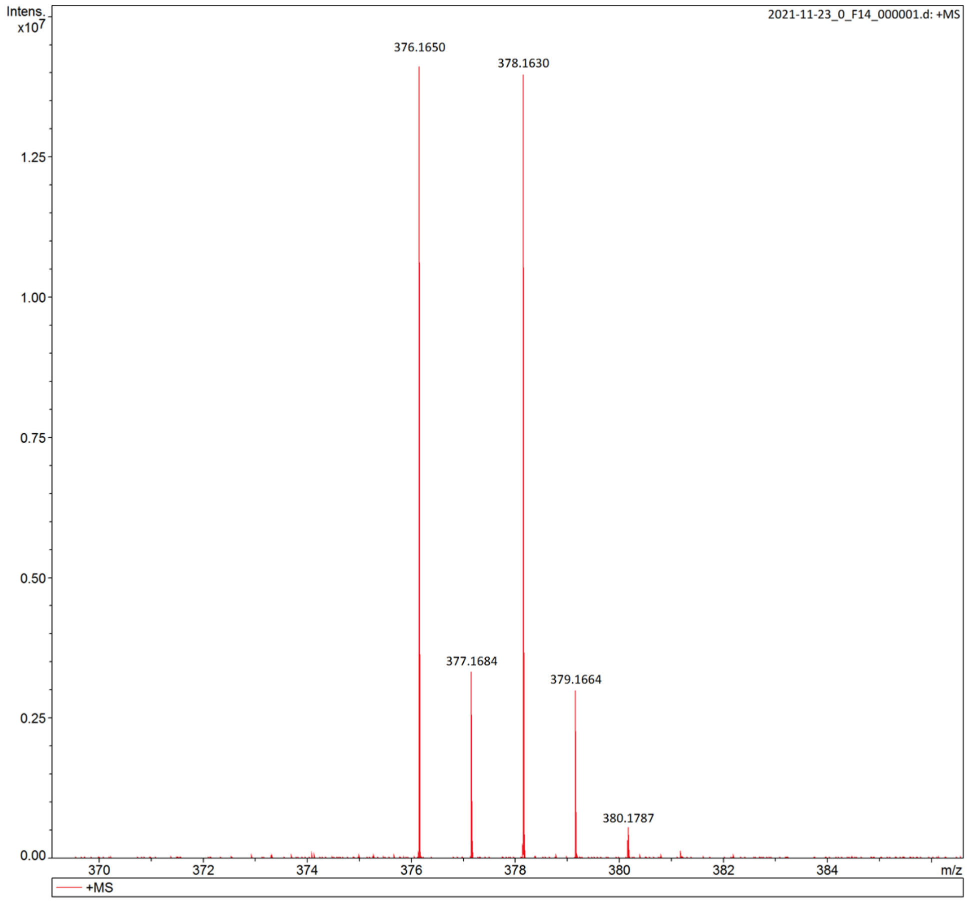

Figure S7. High-resolution MALDI-TOF mass spectrum of compound G_3. The peaks at *m*/*z* 376.1650 corresponds to $[M – Br^-]^+$.

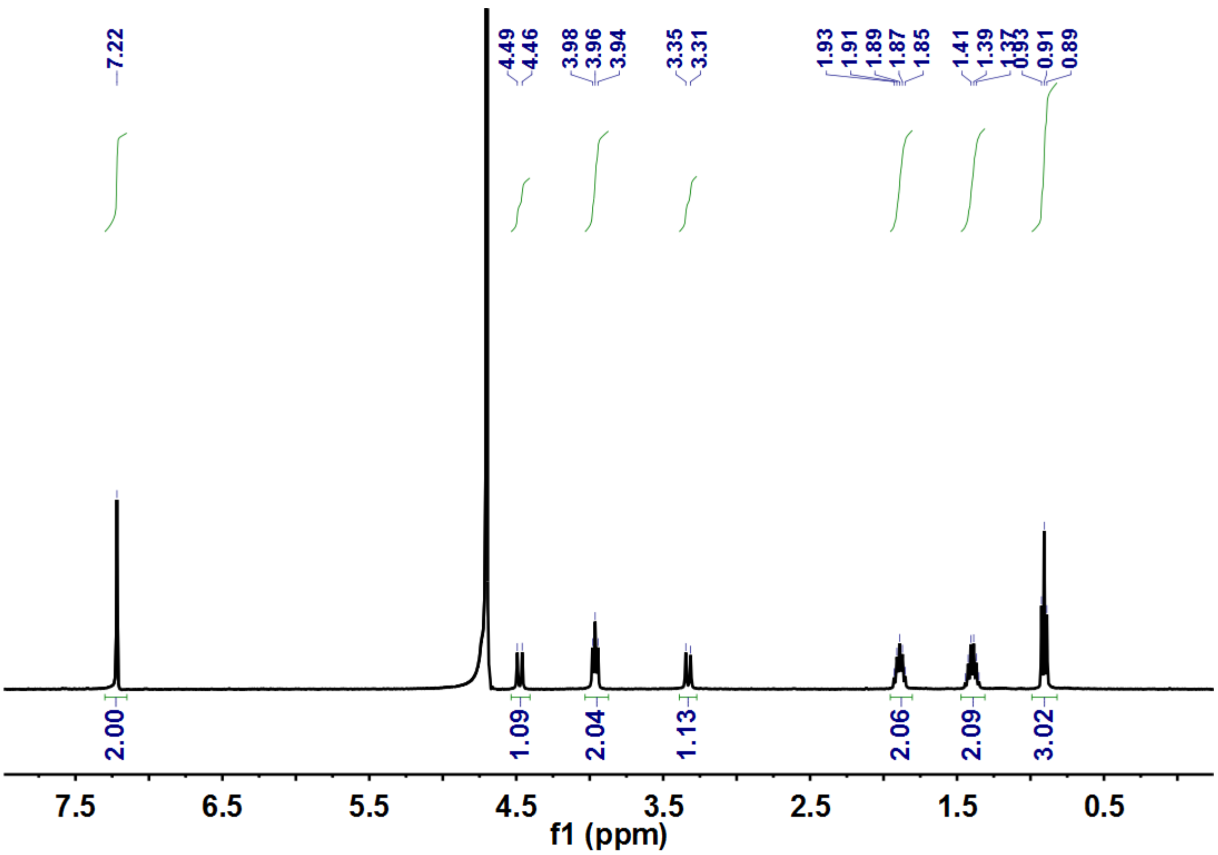

Figure S8. ^{1}H NMR (400 MHz, D_2O, 298 K) spectrum of compound SC4A4.

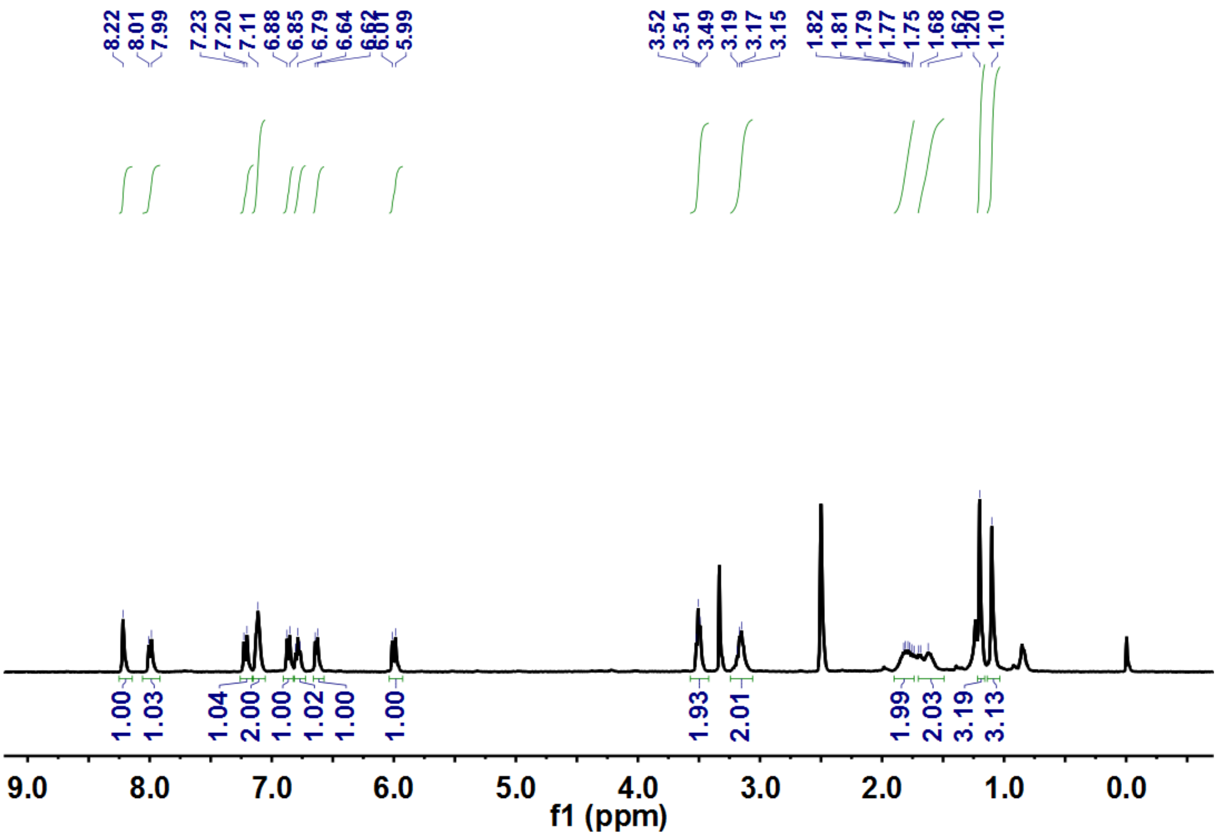

Figure S9. ^{1}H NMR (400 MHz, DMSO-d_6, 298 K) spectrum of compound SP.

3. Investigation of host-guest properties between G_1/G_2 and CB[7]

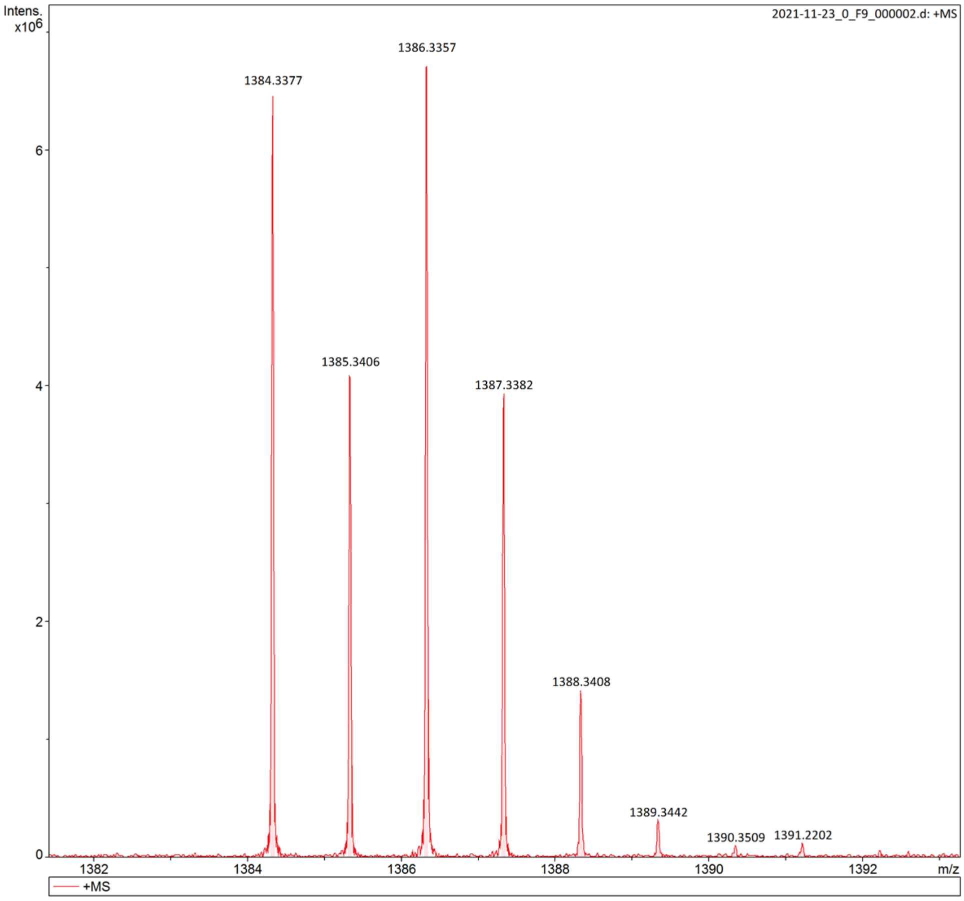

Figure S10. High-resolution MALDI-TOF mass spectrum of compound $G_1 \subset$CB[7]. The peaks at *m/z* 1384.3377 corresponds to $[M - Cl^-]^+$. *m/z* calcd for $C_{52}H_{51}N_{29}O_{14}Br^+$ 1384.3354.

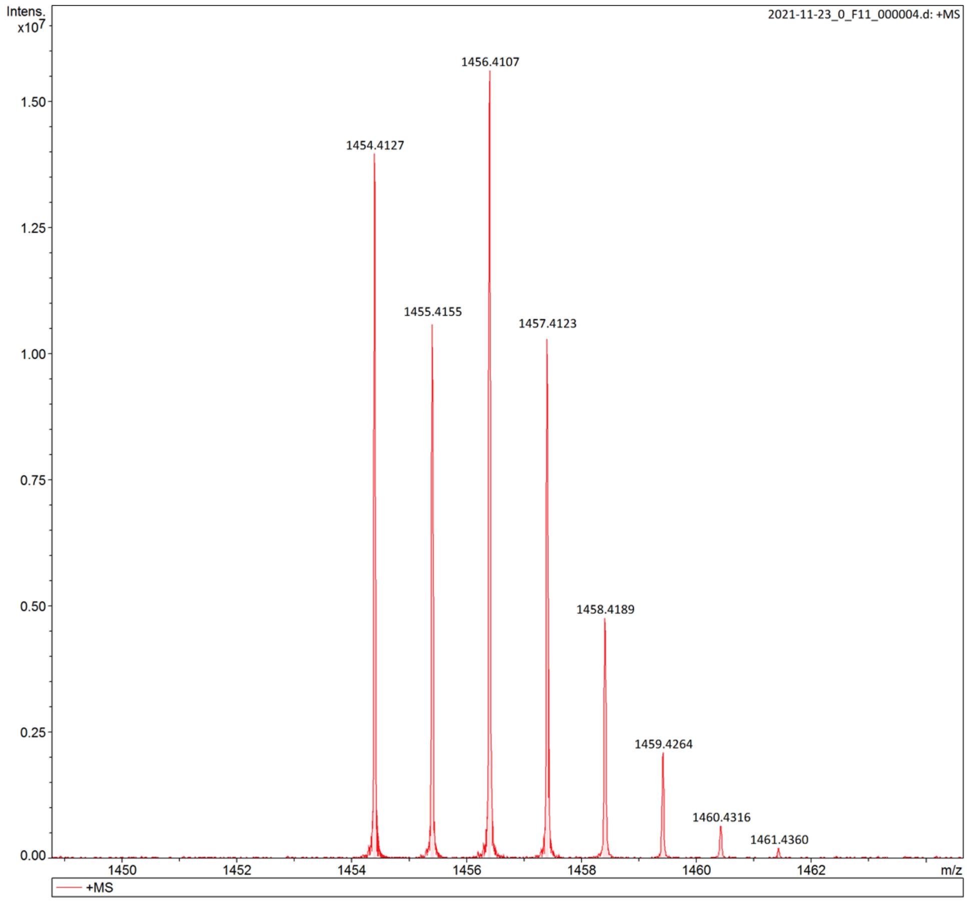

Figure S11. High-resolution MALDI-TOF mass spectrum of compound G$_2$⊂CB[7]. The peaks at *m/z* 1454.4127 corresponds to $[M - Br^-]^+$. *m/z* calcd for $C_{57}H_{61}N_{29}O_{14}Br^+$ 1454.4136.

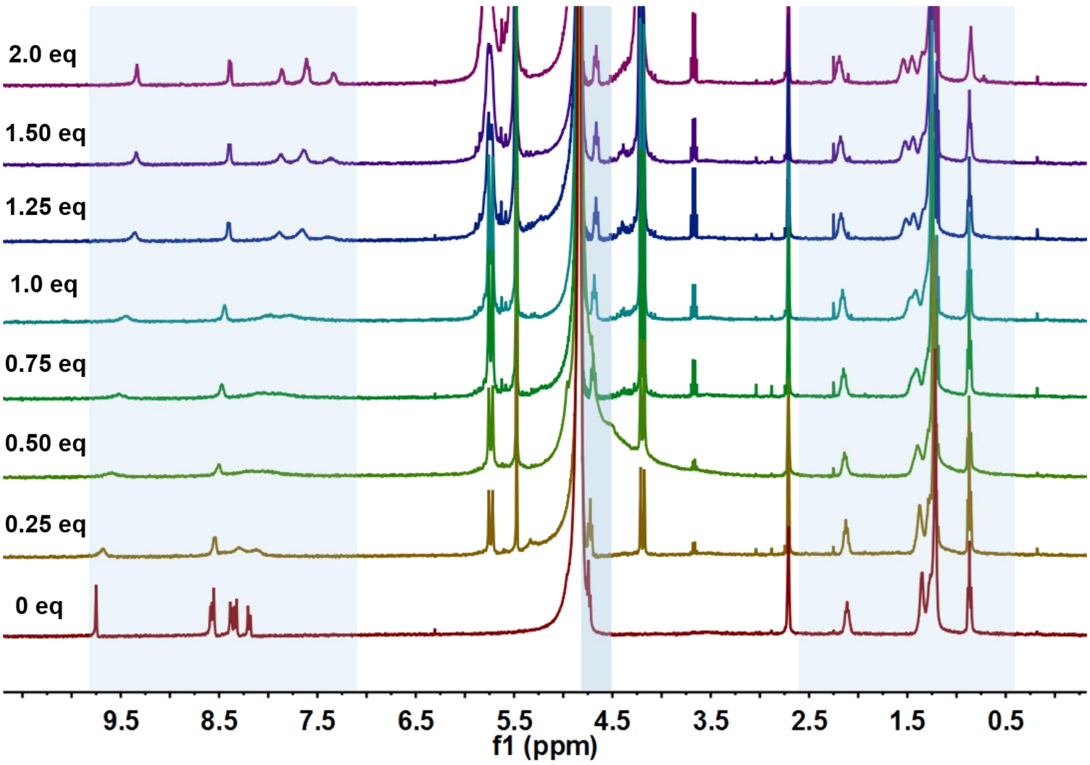

Figure S12. ^{1}H NMR titration spectra (400 MHz, D_2O, 298 K) of G_3 (1.0 mM) in the presence of CB[7] from 0 to 2.0 equivalent.

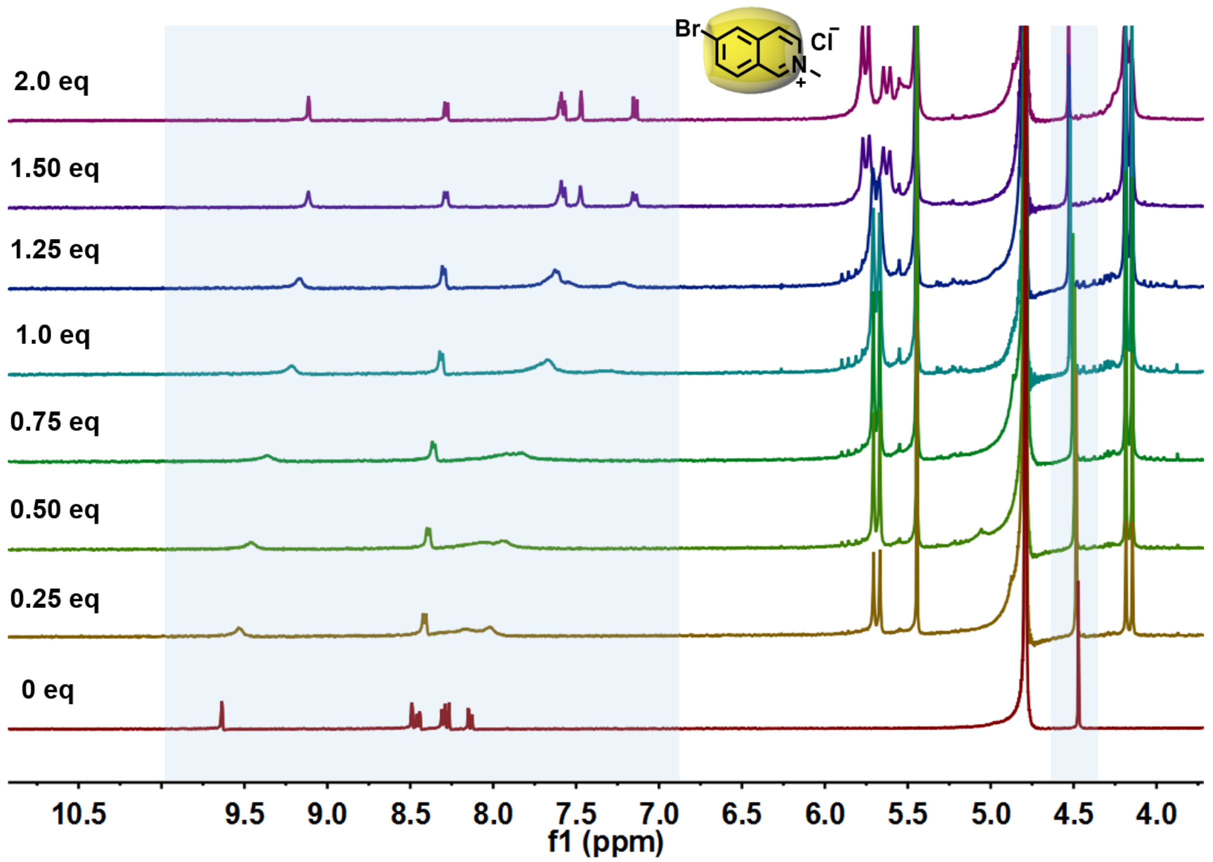

Figure S13. ^{1}H NMR titration spectra (400 MHz, D_2O, 298 K) of G_1 (1.0 mM) in the presence of CB[7] from 0 to 2.0 equivalent.

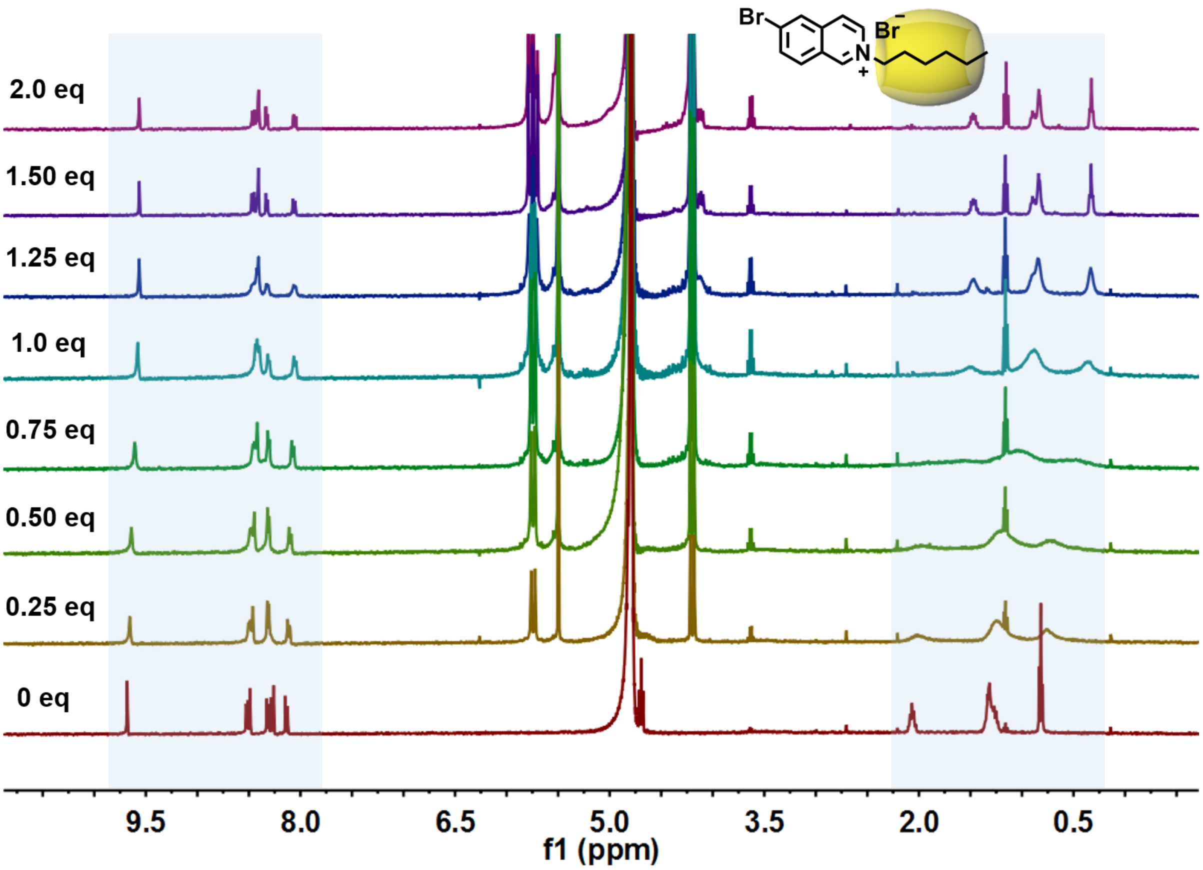

Figure S14. ^{1}H NMR titration spectra (400 MHz, D_2O, 298 K) of G_2 (1.0 mM) in the presence of CB[7] from 0 to 2.0 equivalent.

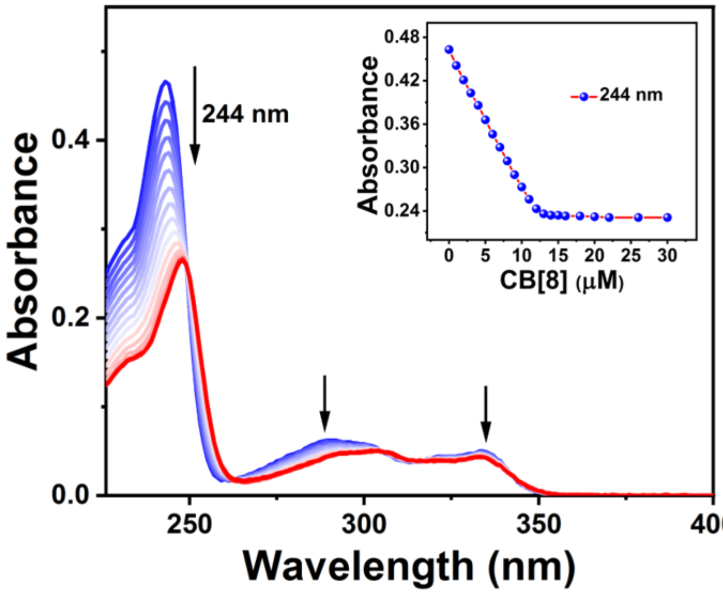

Figure S15. UV−vis absorption spectra and (inset) absorbance intensity changes of G_1 at 244 nm upon addition of CB[7] in H_2O at 298 K ($[G_1] = 1.0 \times 10^{-5}$ M and $[CB[7]] = 0–3.0 \times 10^{-5}$

M).

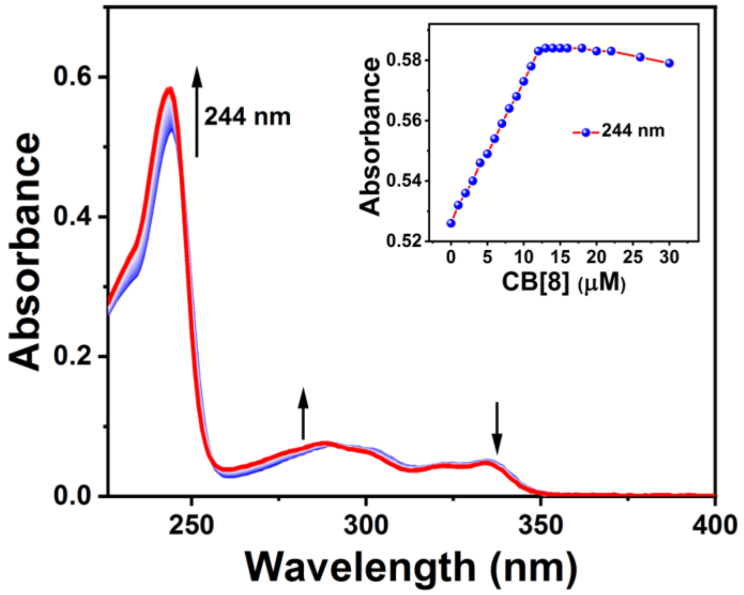

Figure S16. UV−vis absorption spectra and (inset) absorbance intensity changes of G_2 at 244 nm upon addition of CB[7] in H_2O at 298 K ([G_2] = 1.0 × 10^{-5} M and [CB[7]] = 0–3.0 × 10^{-5} M).

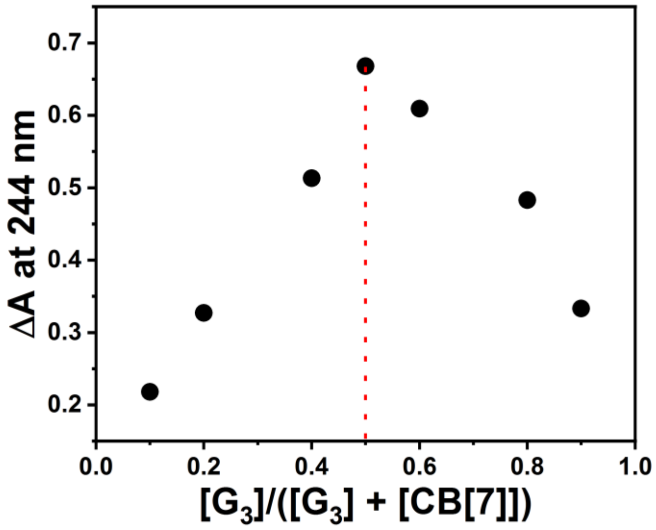

Figure S17. Job plot of G_3 and CB[7] acquired by recording the absorbance at 244 nm at 298 K ([G_3] + [CB[7]] = 5.0 × 10^{-5} M).

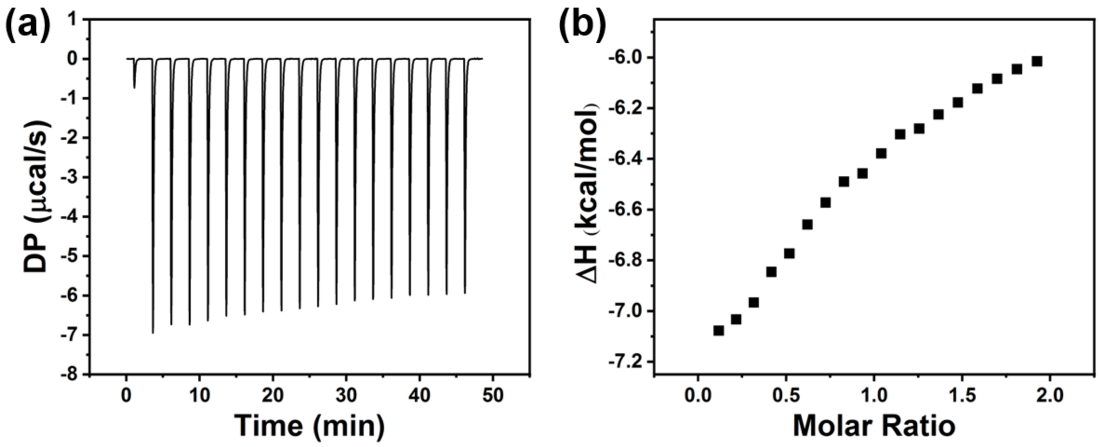

Figure S18. Calorimetric titrations for **$G_3 \subset$CB[7]** system. (a) Raw data and (b) apparent reaction heat ([G_3] (cell) = 0.5 mM, [CB[7]] (syringe) = 5.0 mM, 298 K).

4. Studies on optical behaviors of G_1-G_3 after complexation with CB[7] and cascaded assembly with SC4A4

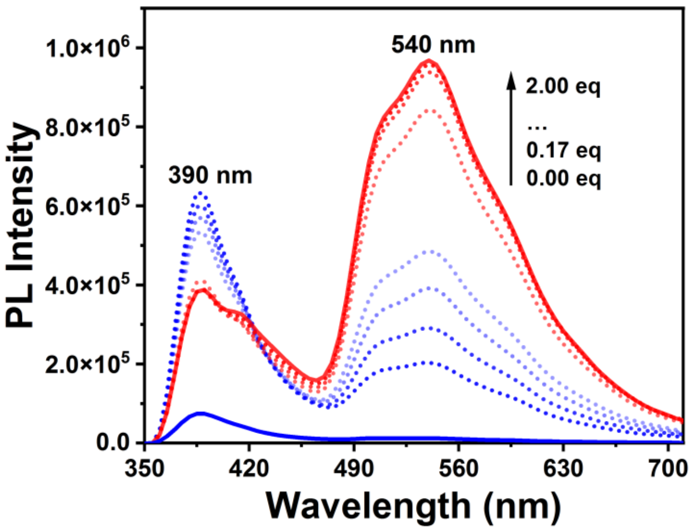

Figure S19. The prompt photoluminescence spectral changes of G_3 upon addition of 0, 0.17, 0.33, 0.50, 0.67, 1.0, 1.33, 1.67 and 2.0 equivalent CB[7] in water at 298 K ([G_3] = 5.0 × 10^{-5} M, λ_{ex} = 300 nm).

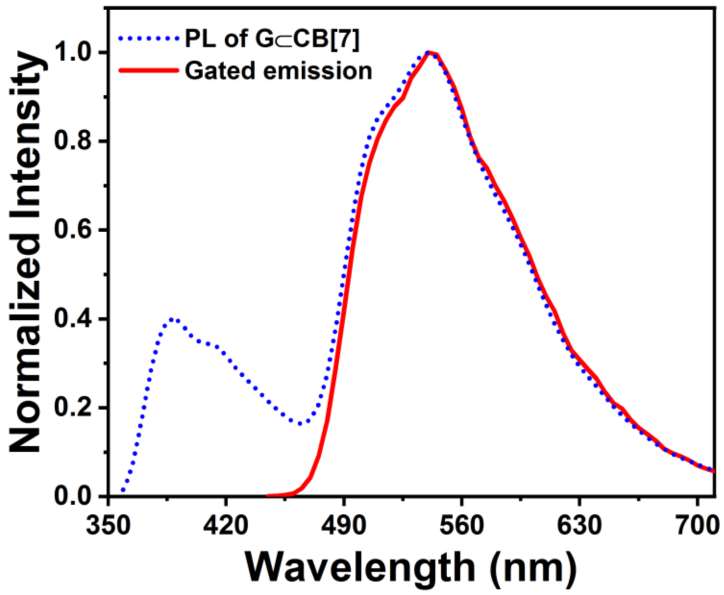

Figure S20. Normalized prompt photoluminescence spectrum and gated emission spectrum (delay 50 μs) of G_3⊂CB[7].

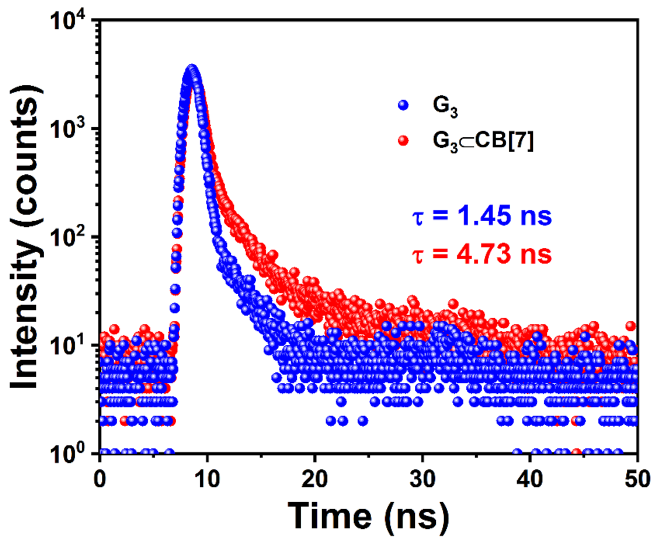

Figure S21. Time-resolved photoluminescence decay spectra of G_3 and G_3⊂CB[7] at 390 nm in water at 298 K.

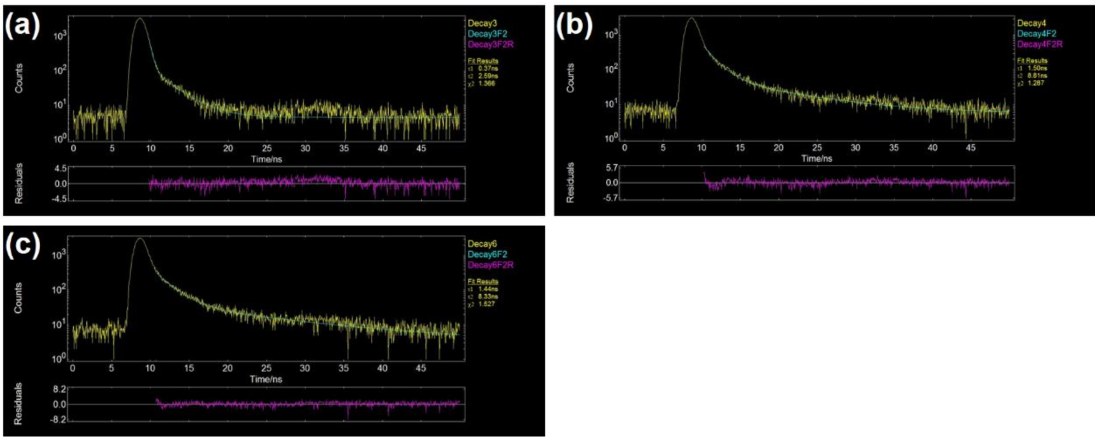

Figure S22. Time-resolved photoluminescence decay fitting curve of (a) G_3, (b) G_3⊂CB[7] and (c) G_3⊂CB[7]@SC4A4 at 390 nm in water at 298 K.

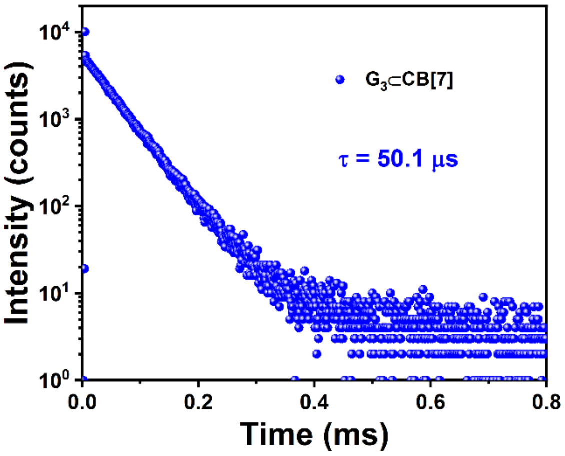

Figure S23. Time-resolved photoluminescence decay spectrum of G_3⊂CB[7] at 540 nm in H_2O at 298 K.

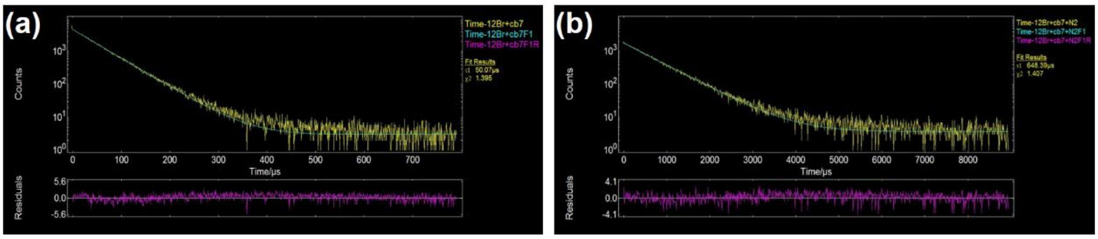

Figure S24. Time-resolved photoluminescence decay fitting curve of G_3⊂CB[7] at 540 nm in water at 298 K under (a) air condition and (b) N_2 atmosphere, respectively.

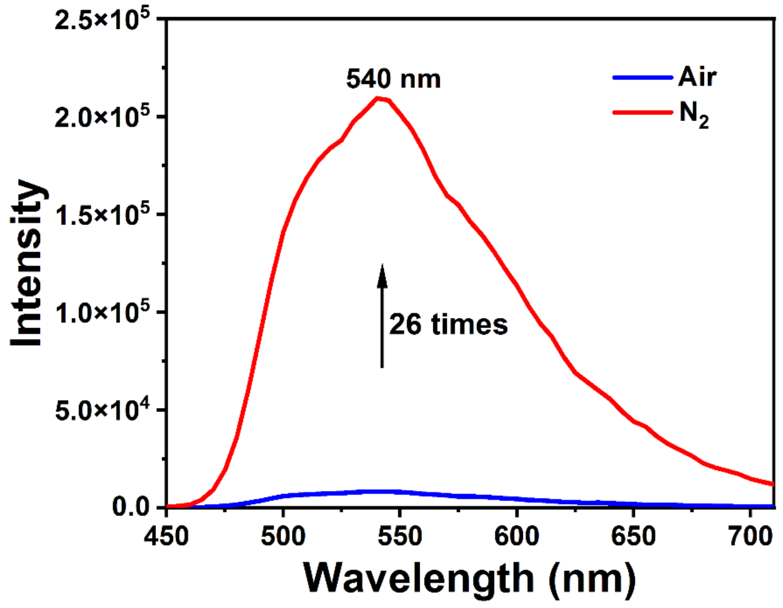

Figure S25. Phosphorescence emission spectra (delay 50 μs) of G_3⊂CB[7]@SC4A4 in water before and after N_2 bubbling at 298 K ([G_3] = 5.0 × 10^{-5} M, [CB[7]] = 7.5 × 10^{-5} M, [SC4A4] = 5.0 × 10^{-5} M, λ_{ex} = 300 nm).

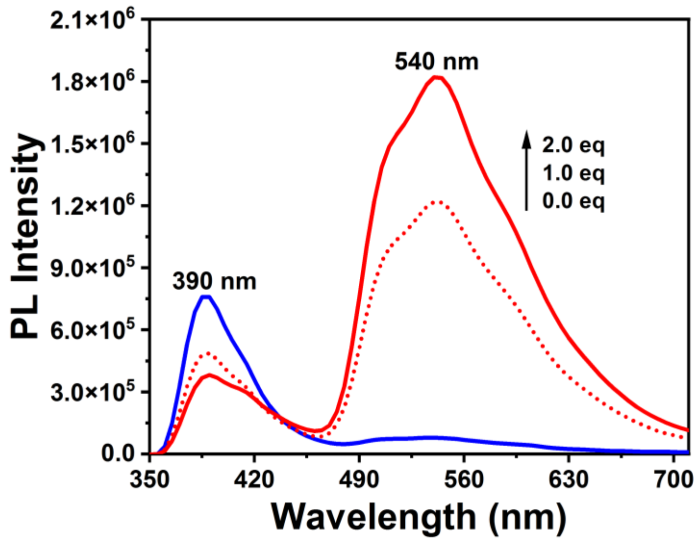

Figure S26. The prompt photoluminescence spectral changes of G_1 upon addition of 0, 1.0 and 2.0 equivalent CB[7] in water at 298 K ([G_1] = 5.0 × 10^{-5} M, λ_{ex} = 300 nm).

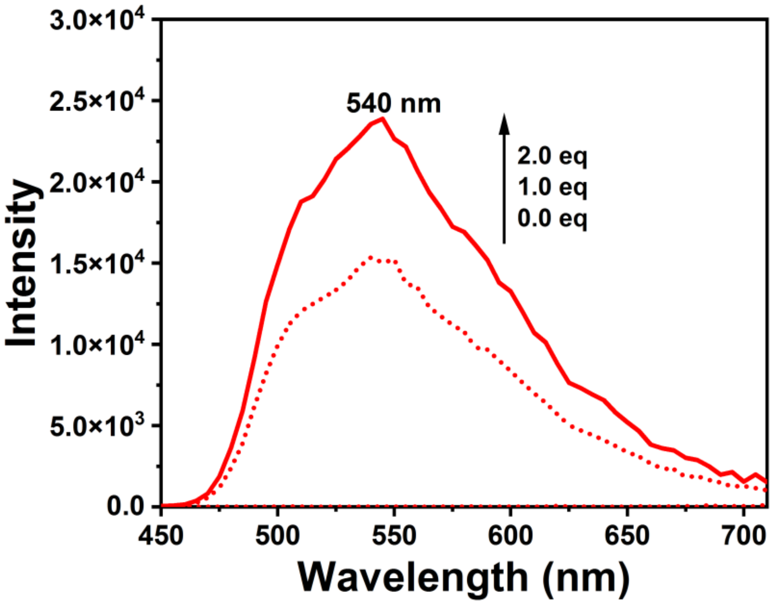

Figure S27. Phosphorescence emission spectra (delay 50 μs) of G_1 upon addition of 0, 1.0 and 2.0 equivalent CB[7] in water at 298 K ($[G_1] = 5.0 \times 10^{-5}$ M, λ_{ex} = 300 nm).

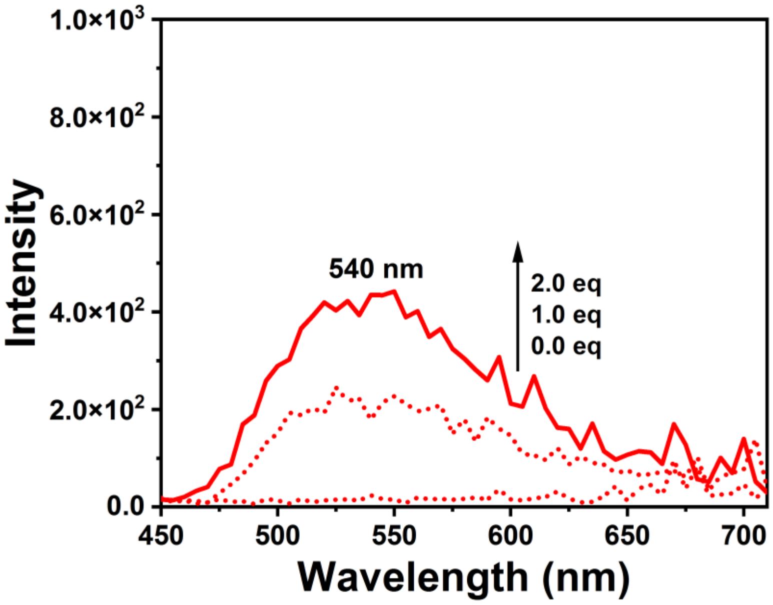

Figure S28. Phosphorescence emission spectra (delay 50 μs) of G_2 upon addition of 0, 1.0 and 2.0 equivalent CB[7] in water at 298 K ($[G_2] = 5.0 \times 10^{-5}$ M, λ_{ex} = 300 nm).

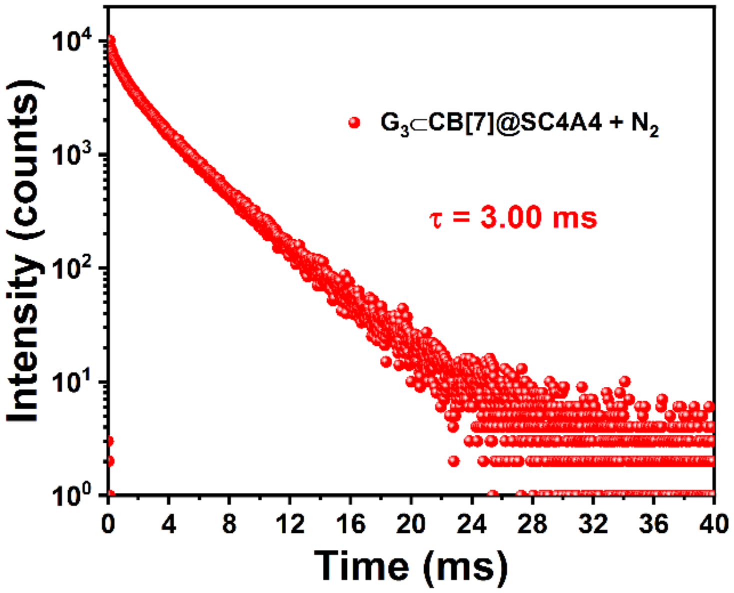

Figure S29. Time-resolved photoluminescence decay spectrum of G_3⊂CB[7]@SC4A4 at 540 nm in H_2O at 298 K under N_2 atmosphere.

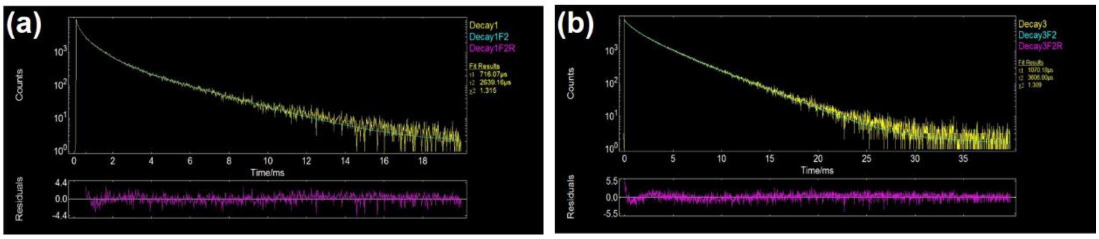

Figure S30. Time-resolved photoluminescence decay fitting curves of G_3⊂CB[7]@SC4A4 at 540 nm in water at 298 K under (a) air condition and (b) N_2 atmosphere.

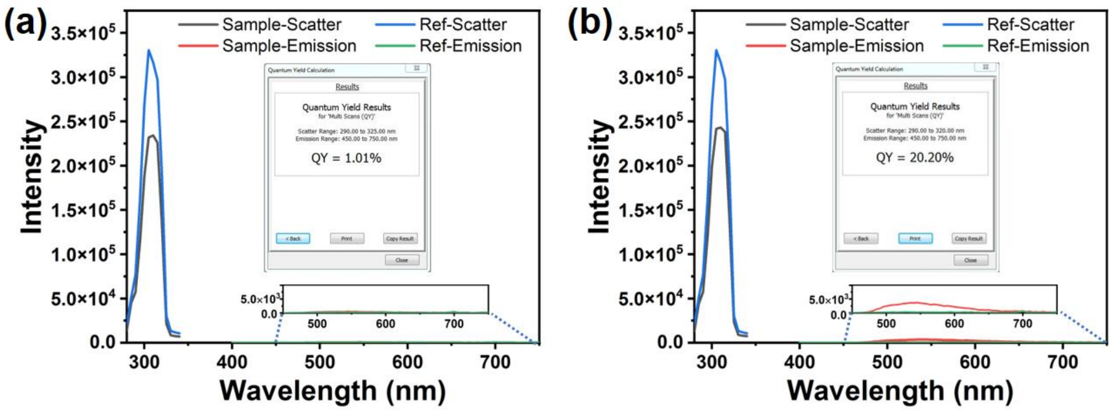

Figure S31. The phosphorescence quantum yields of (a) G_3⊂CB[7], (b) G_3⊂CB[7]@SC4A4 under ambient conditions.

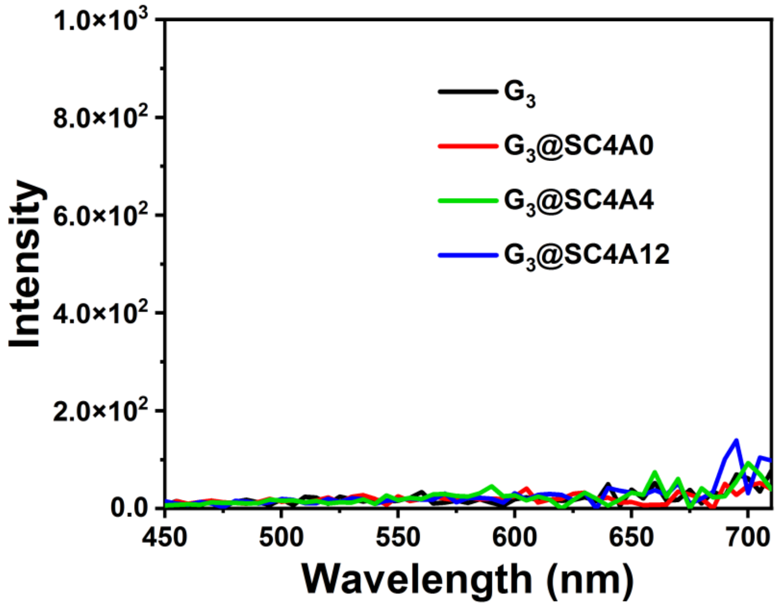

Figure S32. Phosphorescence emission spectra (delay 50 μs) of G_3 upon addition of 1.0 equivalent SC4A0, SC4A4, SC4A12 in water at 298 K ($[G_3] = 5.0 \times 10^{-5}$ M, $\lambda_{ex} = 300$ nm).

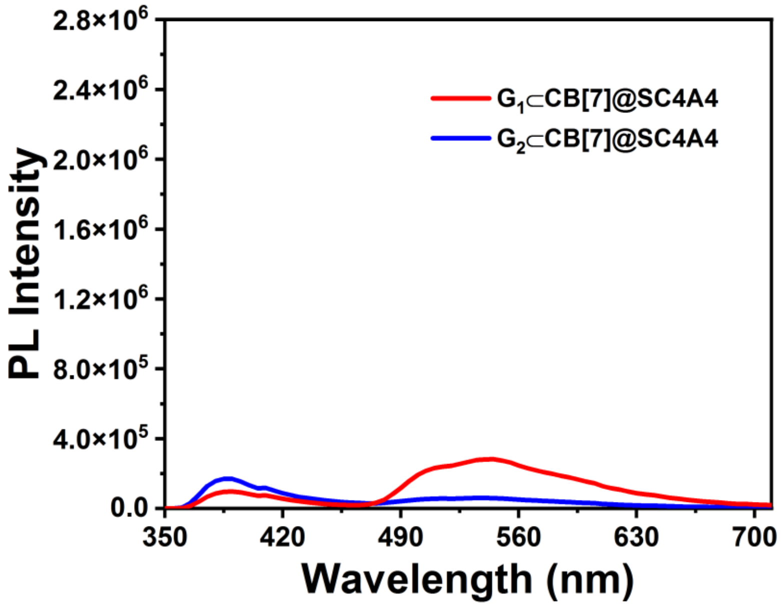

Figure S33. The prompt photoluminescence spectra of $G_1 \subset CB[7]@SC4A4$ and $G_2 \subset CB[7]@SC4A4$ in water at 298 K ($[G_1] = [G_2] = 5.0 \times 10^{-5}$ M, $[CB[7]] = 7.5 \times 10^{-5}$ M, $[SC4A4] = 5.0 \times 10^{-5}$ M, $\lambda_{ex} = 300$ nm).

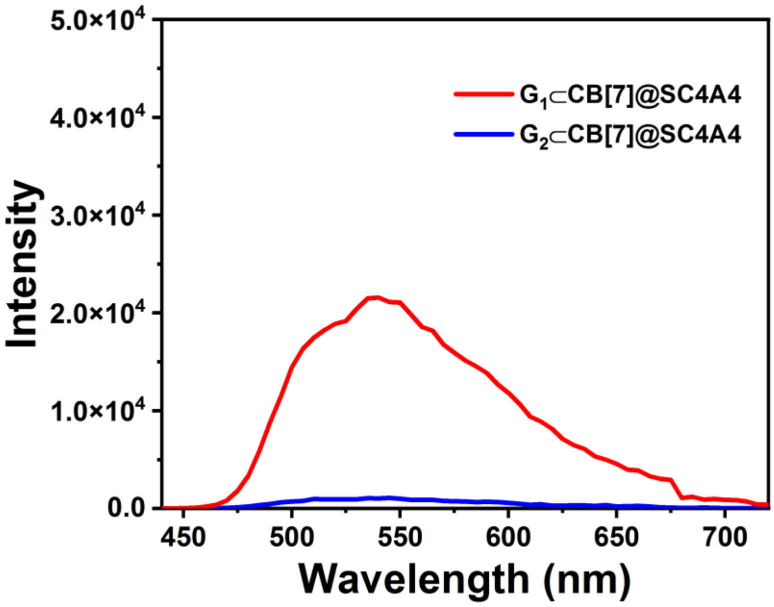

Figure S34. Phosphorescence emission spectra (delay 50 μs) of $G_1 \subset$CB[7]@SC4A4 and $G_2 \subset$CB[7]@SC4A4 in water at 298 K ([G_1] = [G_2] = 5.0×10^{-5} M, [CB[7]] = 7.5×10^{-5} M, [SC4A4] = 5.0×10^{-5} M, λ_{ex} = 300 nm).

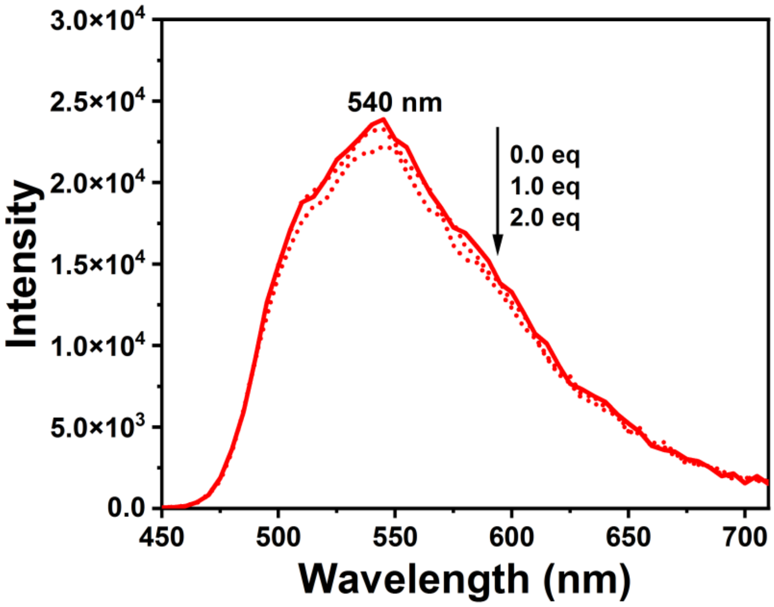

Figure S35. Phosphorescence emission spectral changes (delay 50 μs) of $G_1 \subset$CB[7] upon addition of 0, 1.0 and 2.0 equivalent SC4A4 in water at 298 K ([G_1] = 5.0×10^{-5} M, [CB[7]] = 1.0×10^{-4} M, λ_{ex} = 300 nm).

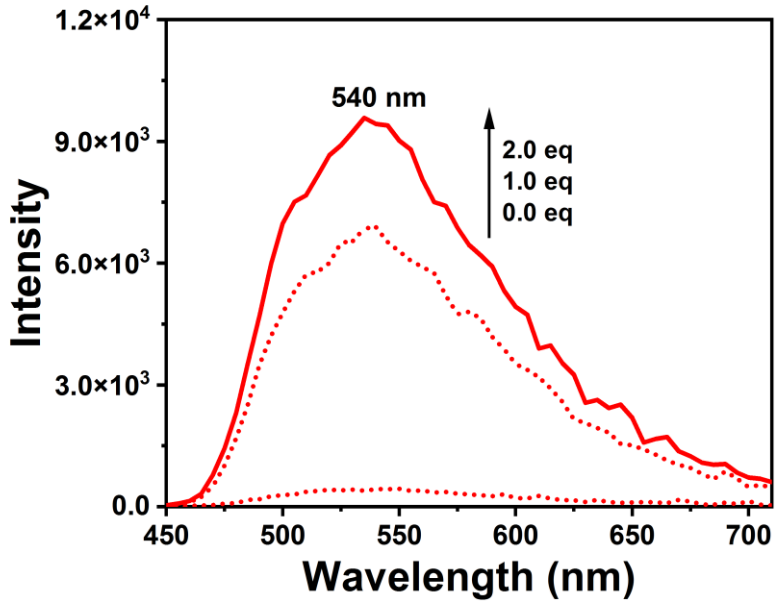

Figure S36. Phosphorescence emission spectral changes (delay 50 μs) of G_2⊂CB[7] upon addition of 0, 1.0 and 2.0 equivalent SC4A4 in water at 298 K ($[G_2] = 5.0 \times 10^{-5}$ M, $[CB[7]] = 1.0 \times 10^{-4}$ M, $\lambda_{ex} = 300$ nm).

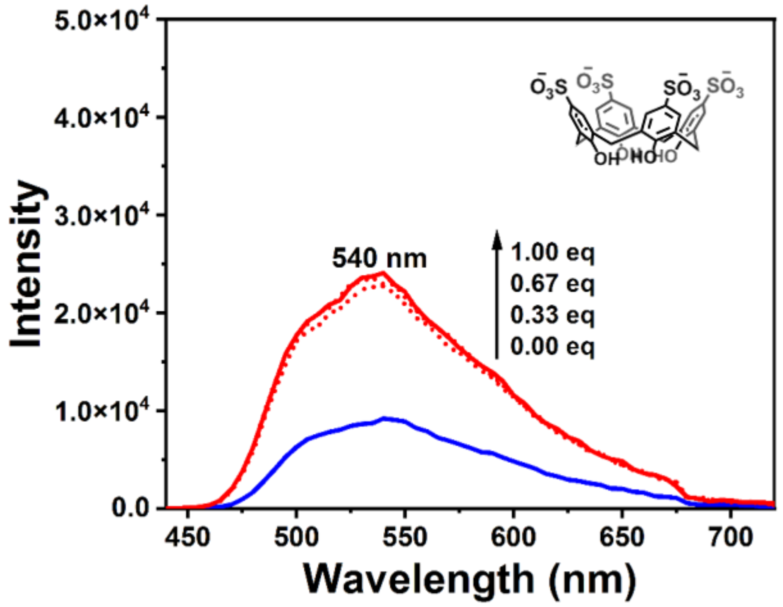

Figure S37. Phosphorescence emission spectral changes (delay 50 μs) of G_3⊂CB[7] upon addition of 0, 0.33, 0.67 and 1.0 equivalent SC4A0 in water at 298 K ($[G_3] = 5.0 \times 10^{-5}$ M, $[CB[7]] = 7.5 \times 10^{-5}$ M, $\lambda_{ex} = 300$ nm).

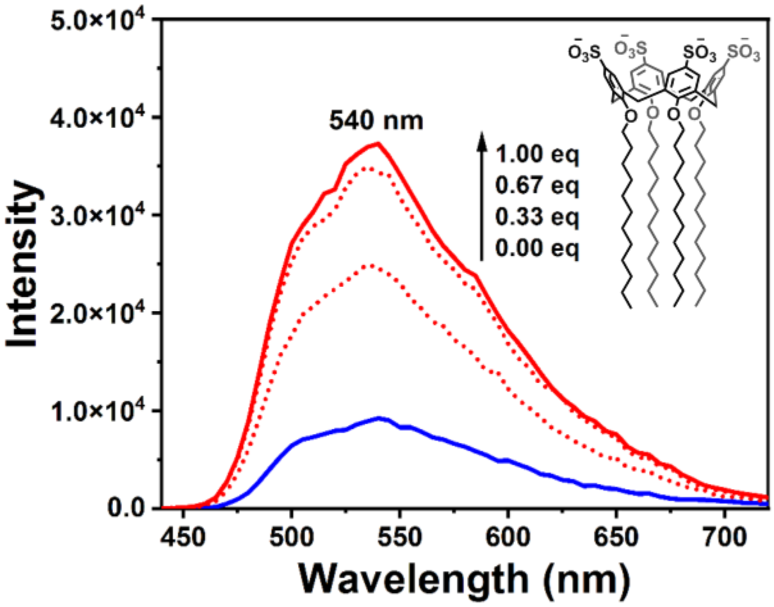

Figure S38. Phosphorescence emission spectral changes (delay 50 μs) of G_3⊂CB[7] upon addition of 0, 0.33, 0.67 and 1.0 equivalent SC4A12 in water at 298 K ($[G_3] = 5.0 \times 10^{-5}$ M, $[CB[7]] = 7.5 \times 10^{-5}$ M, λ_{ex} = 300 nm).

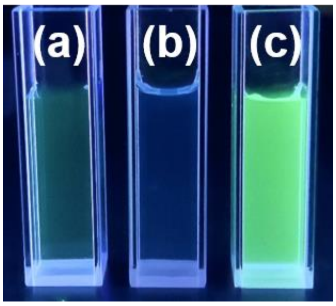

Figure S39. Photographs of (a) G_1⊂CB[7]@SC4A4, (b) G_2⊂CB[7]@SC4A4, and (c) G_3⊂CB[7]@SC4A4 under UV light.

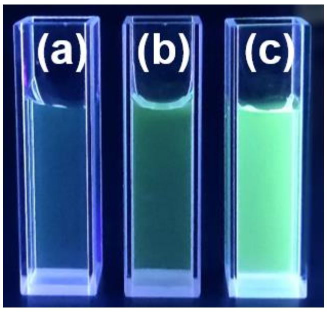

Figure S40. Photographs of (a) G_3⊂CB[7]@SC4A0, (b) G_3⊂CB[7]@SC4A12, and (c) G_3⊂CB[7]@SC4A4 under UV light.

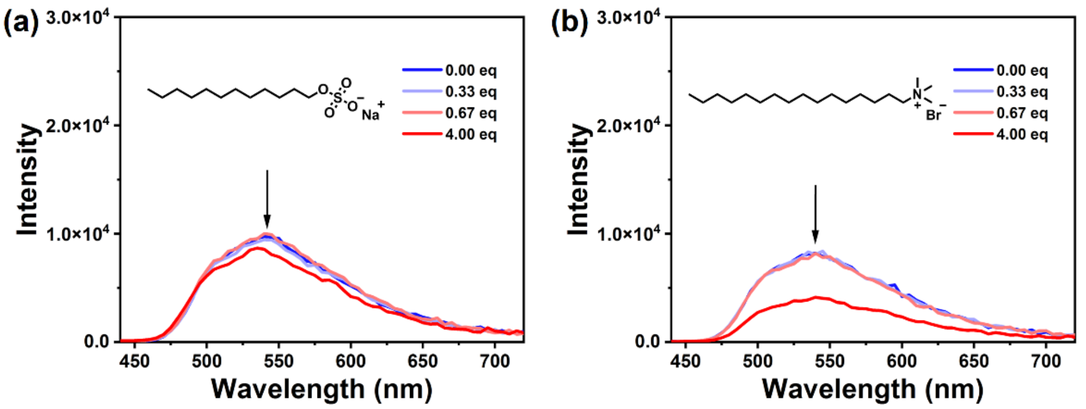

Figure S41. Phosphorescence emission spectral changes (delay 50 μs) of $G_3 \subset CB[7]$ upon addition of 0, 0.33, 0.67 and 4.00 equivalent (a) sodium dodecyl sulfate or (b) cetyltrimethylammonium bromide in water at 298 K ([G_3] = 5.0 × 10^{-5} M, [CB[7]] = 7.5 × 10^{-5} M, λ_{ex} = 300 nm).

5. Characterizations of $G_3 \subset$CB[7]@SC4A4 and $G_3 \subset$CB[7]@SC4A4/SP assembly

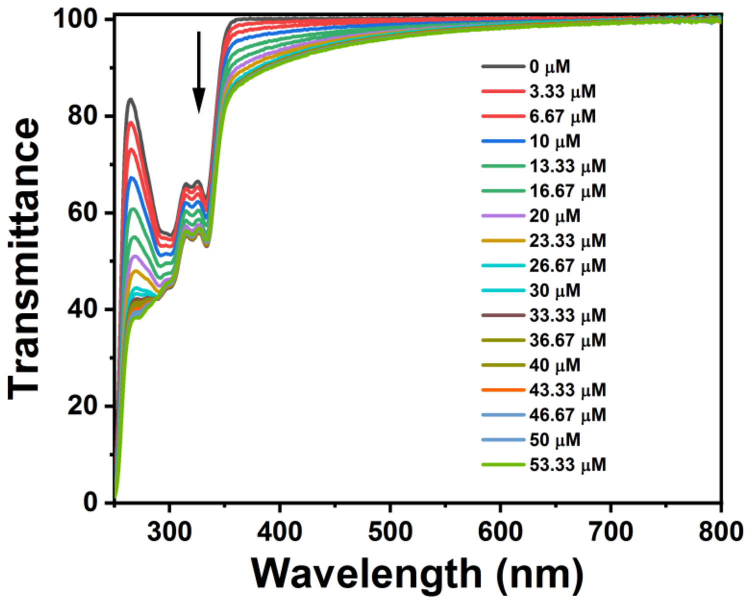

Figure S42. Optical transmittance of $G_3 \subset CB[7]$ ([G_3] = 5.0 × 10^{-5} M, [CB[7]] = 7.5 × 10^{-5} M) upon increasing the concentration of SC4A4 in water at 298 K.

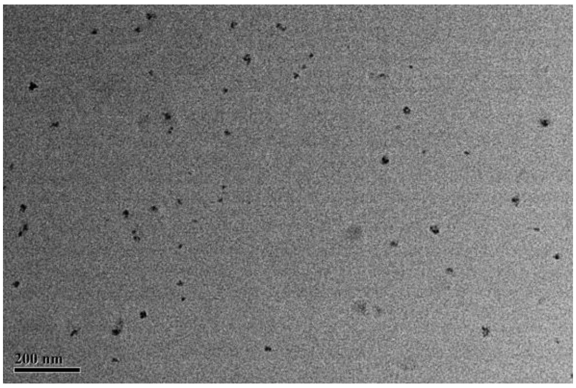

Figure S43. Transmission electron microscopy image of $G_3 \subset CB[7]$.

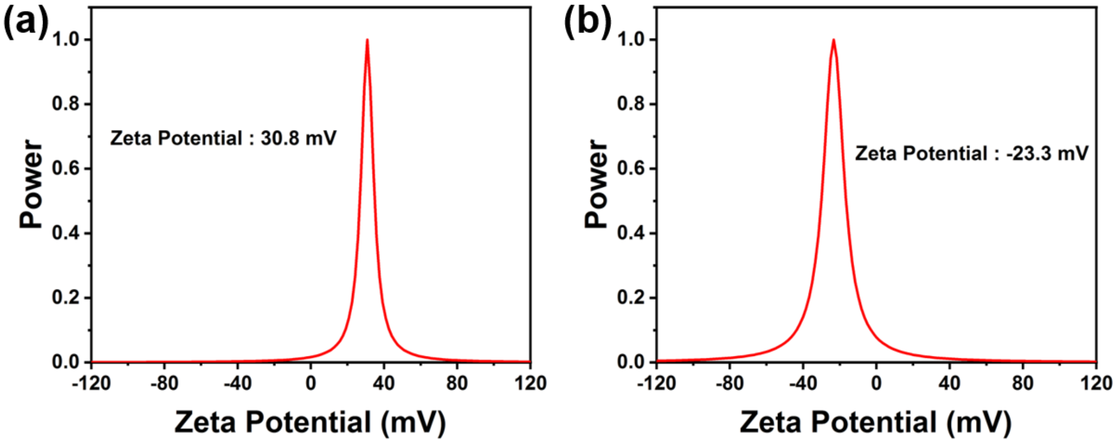

Figure S44. Zeta potential of $G_3 \subset CB[7]$ and $G_3 \subset CB[7]$@SC4A4.

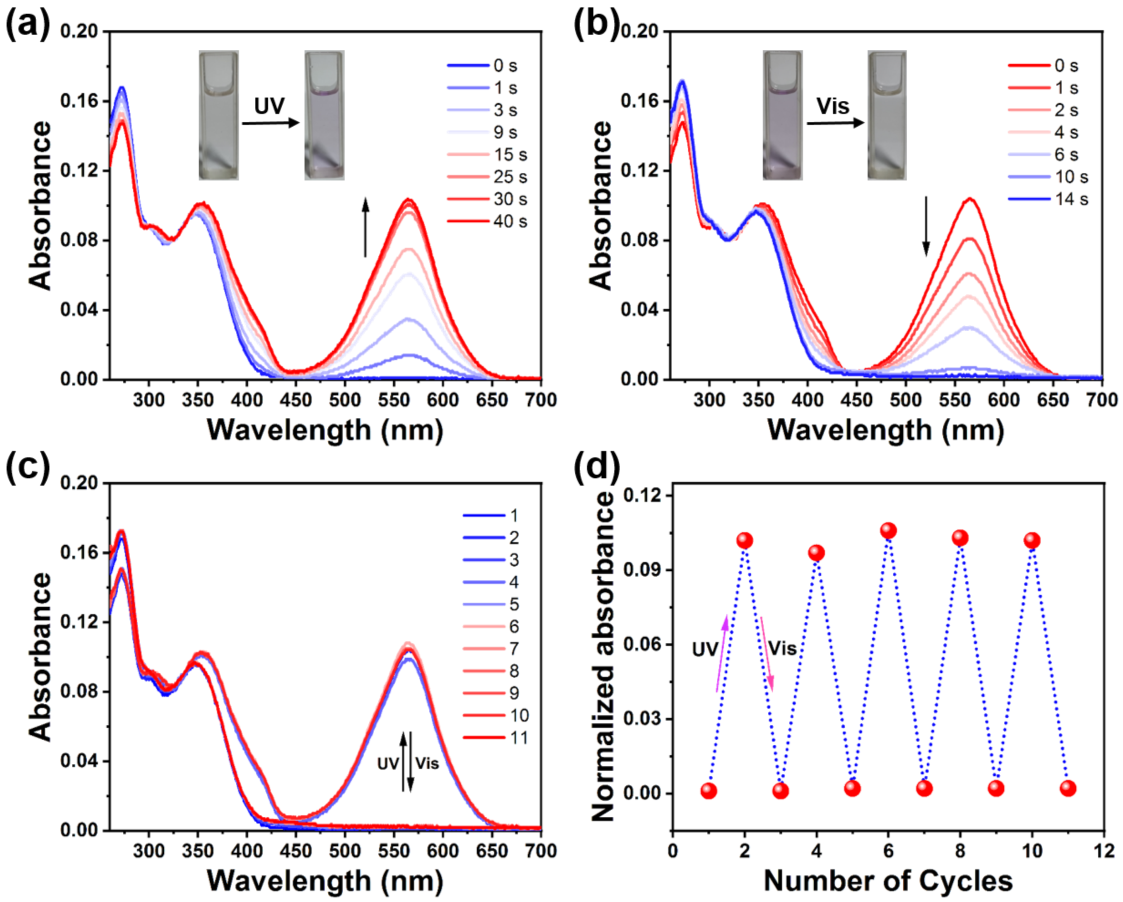

Figure S45. (a) UV−vis absorption spectral changes of SP upon irradiation with UV light in DMSO at 298 K. (b) UV−vis absorption spectral changes of SP upon irradiation with Visible light in DMSO at 298 K. (c) UV−vis absorption spectra of SP upon alternating irradiation with UV and visible light. (d) UV−vis absorption intensity changes of SP at 560 nm upon alternating irradiation with UV and visible light ([SP] = 1.0×10^{-5} M).

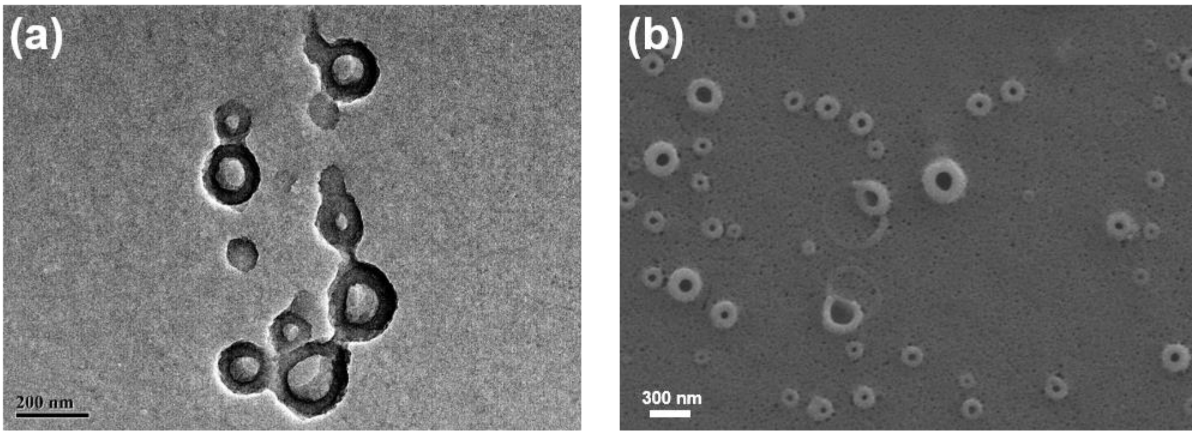

Figure S46. (a) TEM and (b) SEM image of G_3⊂CB[7]@SC4A4/SP.

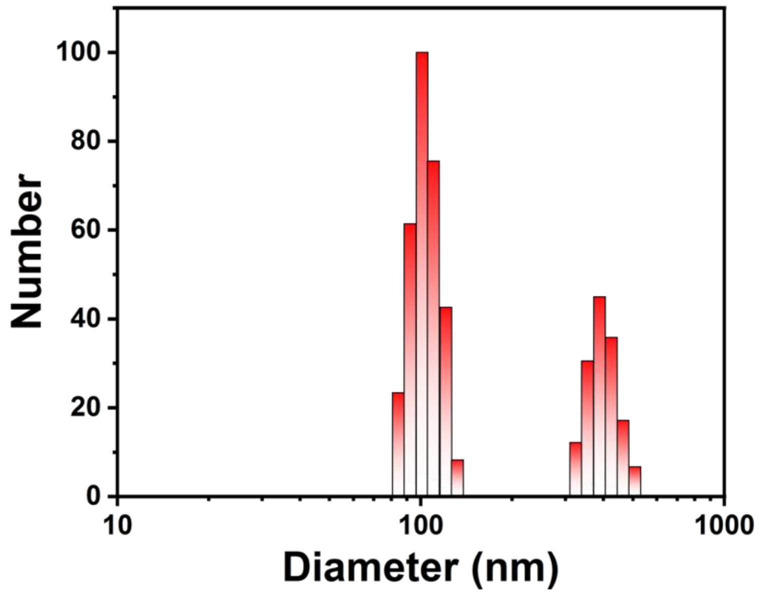

Figure S47. Dynamic light scattering analysis of $G_3 \subset CB[7]@SC4A4/SP$.

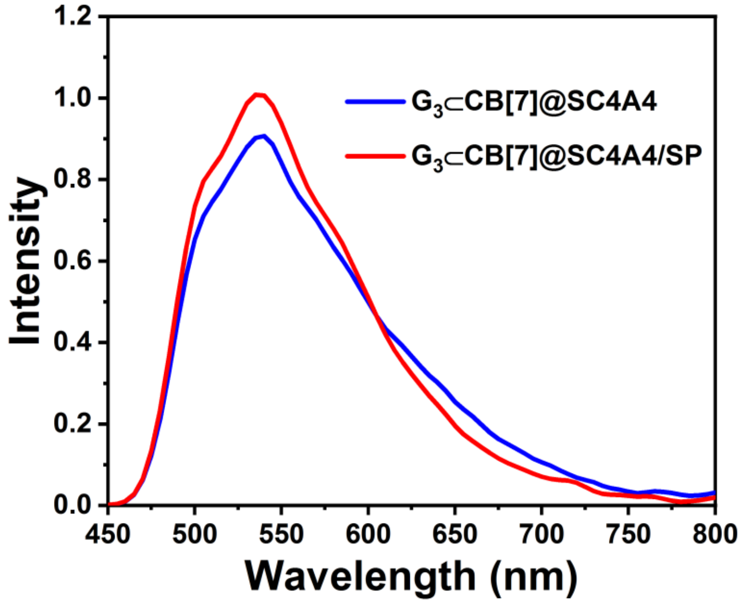

Figure S48. Phosphorescence emission spectra (delay 50 μs) of $G_3 \subset CB[7]$ @SC4A4 and $G_3 \subset CB[7]$ @SC4A4/SP in water at 298 K ($[G_3]$ = [SC4A4] = [SP] = 5.0×10^{-5} M, [CB[7]] = 7.5×10^{-5} M, λ_{ex} = 300 nm).

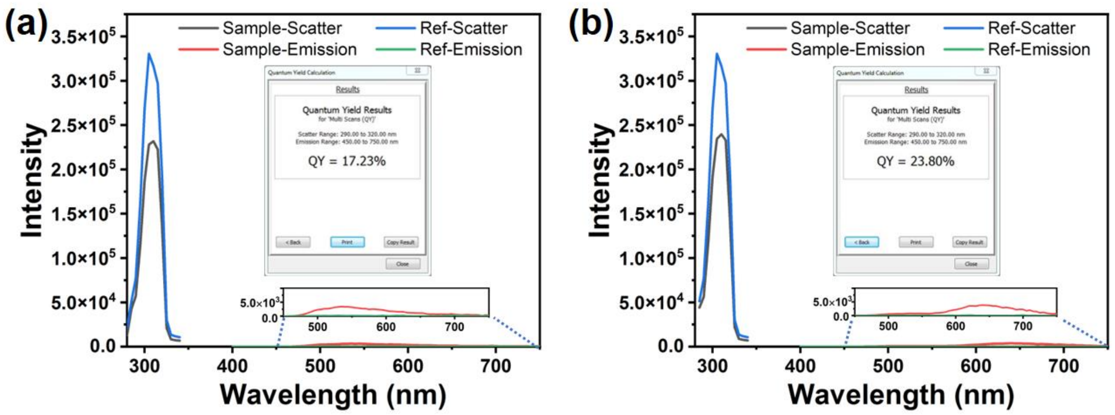

Figure S49. The phosphorescence quantum yields of (a) G_3⊂CB[7]@SC4A4/SP and (b) G_3⊂CB[7]@SC4A4/MC under ambient conditions.

6. Phosphorescence energy transfer measurements of G_3⊂CB[7]@SC4A4/SP

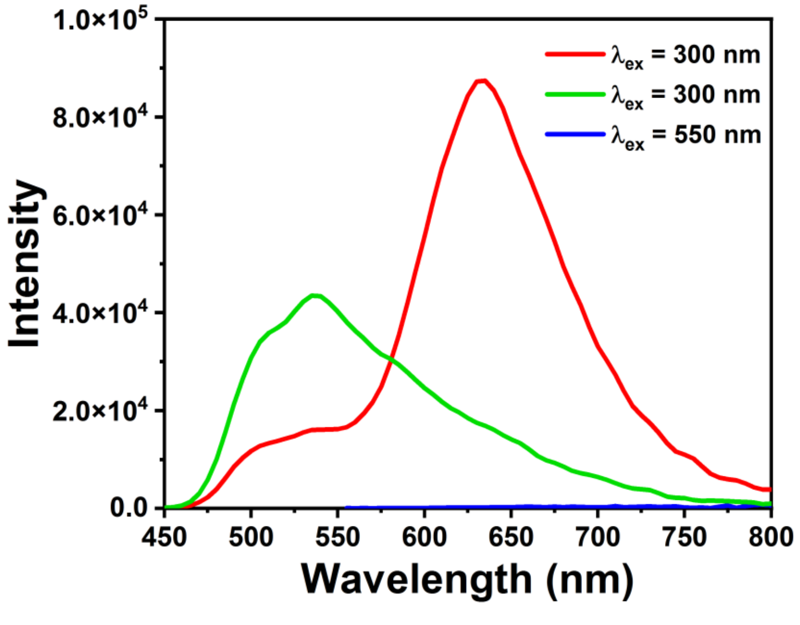

Figure S50. Phosphorescence emission spectra (delay 50 μs) of G_3⊂CB[7]@SC4A4/SP before (green, λ_{ex} = 300 nm) and after UV irradiation (red, λ_{ex} = 300 nm; blue, λ_{ex} = 550 nm) in water at 298 K ([G_3] = 5.0 × 10^{-5} M, [CB[7]] = 7.5 × 10^{-5} M, [SC4A4] = 5.0 × 10^{-5} M, [SP] = 5.0 × 10^{-5} M).

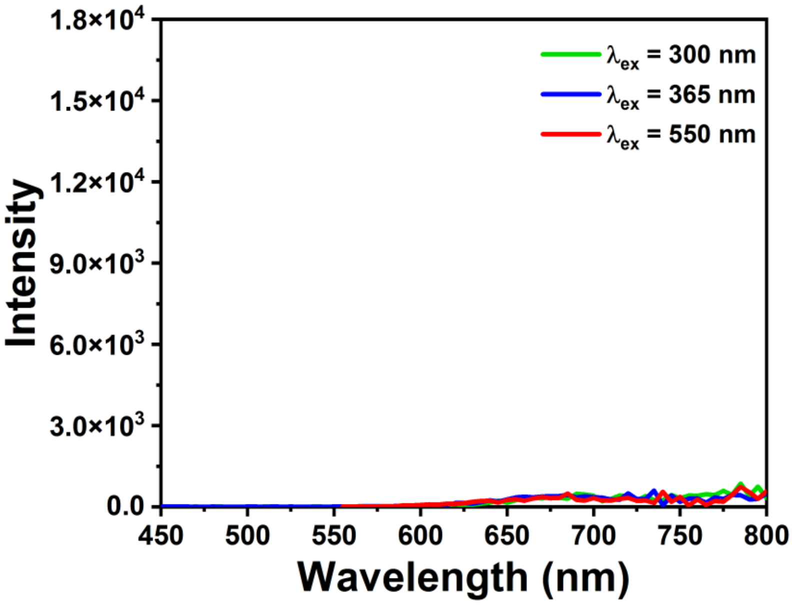

Figure S51. Phosphorescence emission spectra (delay 50 μs) of G_3-free assembly of SP after UV irradiation (red, λ_{ex} = 300 nm; blue, λ_{ex} = 550 nm) in water at 298 K ([SP] = 5.0 × 10^{-5} M).

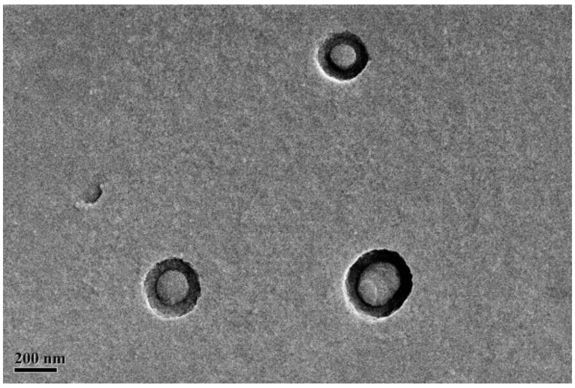

Figure S52. TEM image of G_3⊂CB[7]@SC4A4/MC.

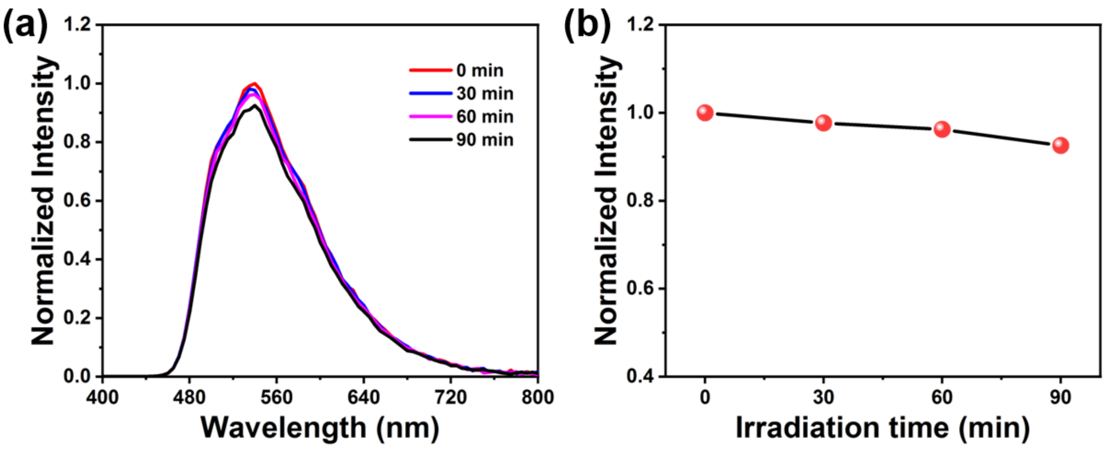

Figure S53. Phosphorescence emission spectral changes (delay 50 μs) of G_3⊂CB[7]@SC4A4 upon irradiation with UV light (365 nm) at various intervals in water at 298 K ([G_3] = 5.0 × 10^{-5} M, [CB[7]] = 7.5 × 10^{-5} M, [SC4A4] = 5.0 × 10^{-5} M).

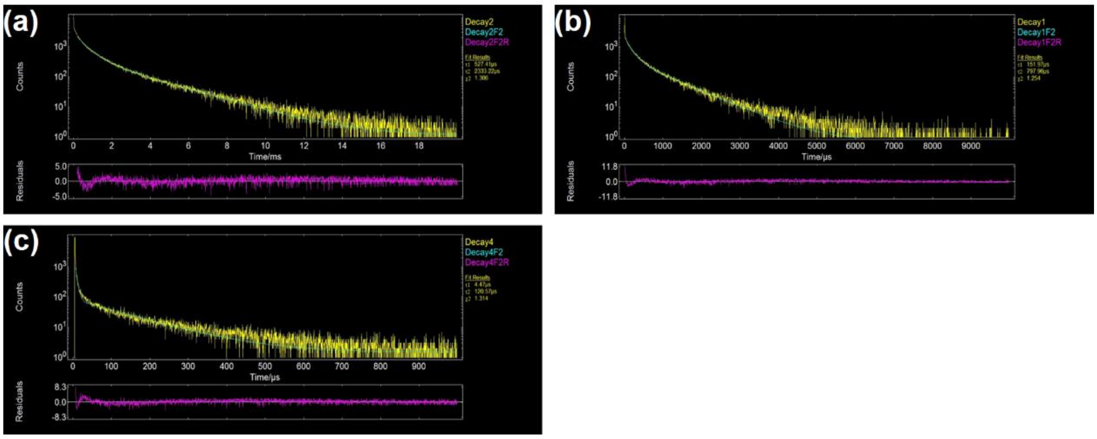

Figure S54. Time-resolved photoluminescence decay fitting curves of G_3⊂CB[7]@SC4A4/SP at 540 nm (a) before and (b) after irradiation with UV light in water at 298 K. (c) Time-resolved photoluminescence decay fitting curve of G_3⊂CB[7]@SC4A4/SP at 635 nm after irradiation with UV light in water at 298 K ([G_3] = [SC4A4] = [SP] = 5.0 × 10^{-5} M, [CB[7]] = 7.5 × 10^{-5} M, λ_{ex} = 300 nm).

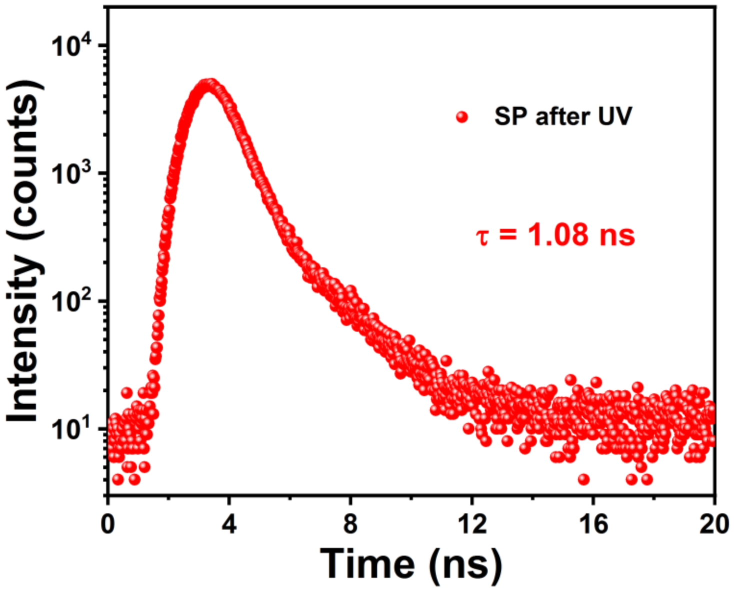

Figure S55. Time-resolved photoluminescence decay spectra of MC at 635 nm at 298 K.

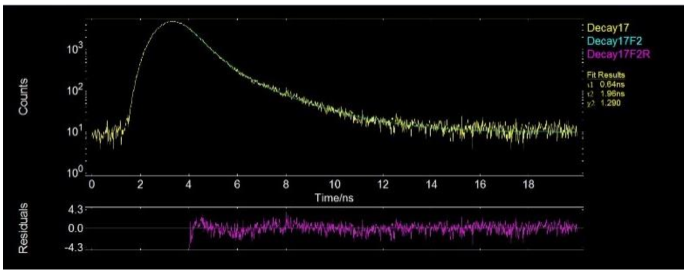

Figure S56. Time-resolved photoluminescence decay fitting curve of MC at 635 nm at 298 K.

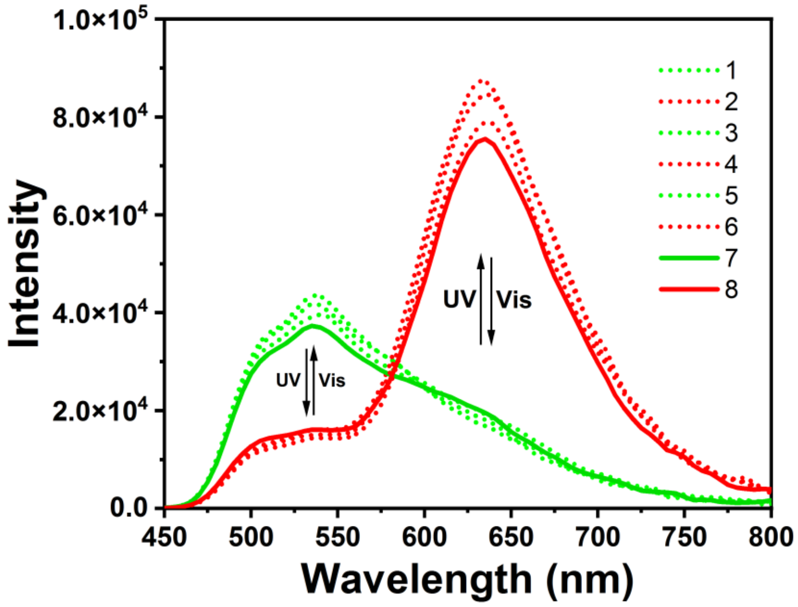

Figure S57. Phosphorescence emission spectra (delay 50 μs) of G_3⊂CB[7]@SC4A4/SP upon alternating irradiation with UV (365 nm, 56 s) and visible light (>420 nm, 10 s) in water at 298 K ([G_3] = 5.0×10^{-5} M, [CB[7]] = 7.5×10^{-5} M, [SC4A4] = 5.0×10^{-5} M, [SP] = 5.0×10^{-5} M).

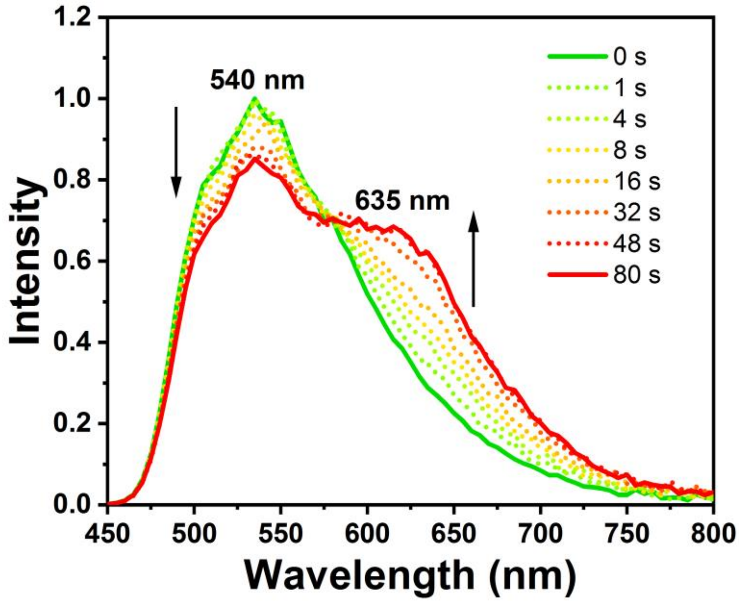

Figure S58. Normalized phosphorescence emission spectral changes (delay 50 μs) of G_3⊂CB[7]@SC4A4/SP upon irradiation with UV light (365 nm, 80 s) in water at 298 K ([G_3] = 5.0 × 10^{-5} M, [CB[7]] = 7.5 × 10^{-5} M, [SC4A4] = 5.0 × 10^{-5} M, [SP] = 8.3 × 10^{-6} M, λ_{ex} = 300 nm).

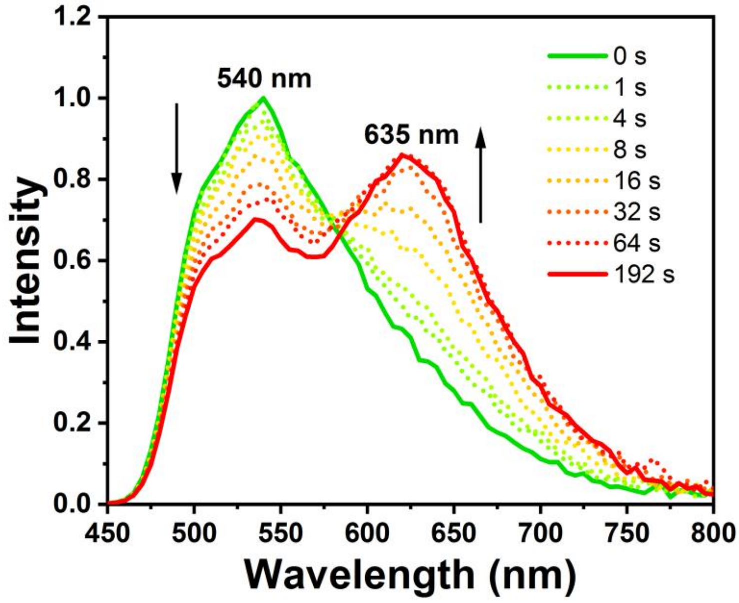

Figure S59. Normalized phosphorescence emission spectral changes (delay 50 μs) of G_3⊂CB[7]@SC4A4/SP upon irradiation with UV light (365 nm, 192 s) in water at 298 K ([G_3] = 5.0 × 10^{-5} M, [CB[7]] = 7.5 × 10^{-5} M, [SC4A4] = 5.0 × 10^{-5} M, [SP] = 2.5 × 10^{-5} M, λ_{ex} =

300 nm).

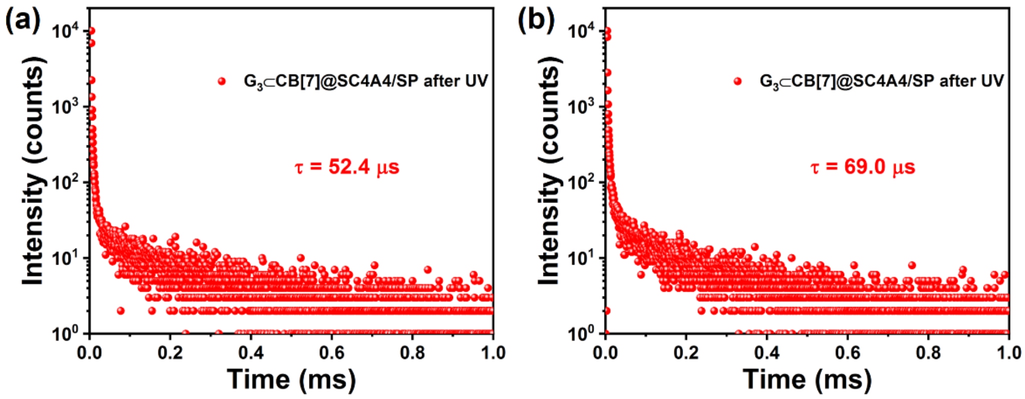

Figure S60. (a) Time-resolved photoluminescence decay spectrum of G_3⊂CB[7]@SC4A4/SP at 635 nm upon irradiation with UV light (365 nm, 80 s) in water at 298 K ([G_3] = 5.0 × 10^{-5} M, [CB[7]] = 7.5 × 10^{-5} M, [SC4A4] = 5.0 × 10^{-5} M, [SP] = 8.3 × 10^{-6} M, λ_{ex} = 300 nm). (b) Time-resolved photoluminescence decay spectrum of G_3⊂CB[7]@SC4A4/SP at 635 nm upon irradiation with UV light (365 nm, 192 s) in water at 298 K ([G_3] = 5.0 × 10^{-5} M, [CB[7]] = 7.5 × 10^{-5} M, [SC4A4] = 5.0 × 10^{-5} M, [SP] = 2.5 × 10^{-5} M, λ_{ex} = 300 nm).

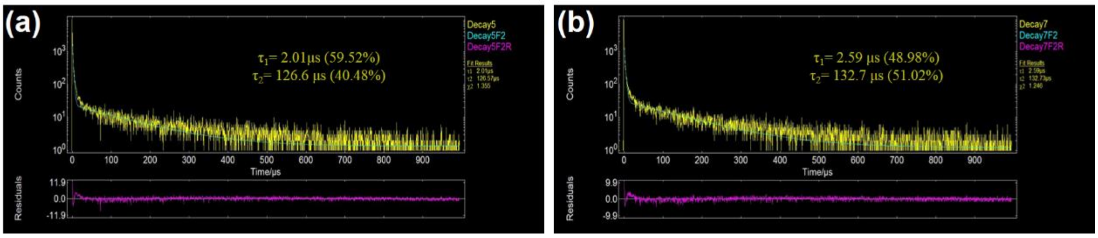

Figure S61. Time-resolved photoluminescence decay fitting curves of G_3⊂CB[7]@SC4A4/SP with different amounts of SP at 635 nm after irradiation with UV light in water at 298 K. (a) [G_3] = 5.0 × 10^{-5} M, [CB[7]] = 7.5 × 10^{-5} M, [SC4A4] = 5.0 × 10^{-5} M, [SP] = 8.3 × 10^{-6} M, λ_{ex} = 300 nm; (b) [G_3] = 5.0 × 10^{-5} M, [CB[7]] = 7.5 × 10^{-5} M, [SC4A4] = 5.0 × 10^{-5} M, [SP] = 2.5 × 10^{-5} M, λ_{ex} = 300 nm (the average lifetime: $\tau = \tau_1 \times Rel_1\% + \tau_2 \times Rel_2\%$).

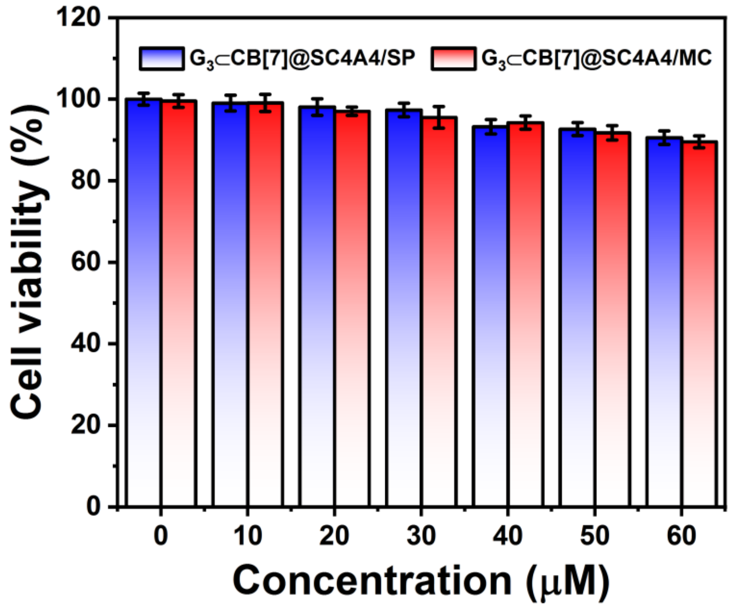

Figure S62. Cell viability of A549 cancer cells after 24 h incubation with G_3⊂CB[7]@SC4A4/SP and G_3⊂CB[7]@SC4A4/MC. n = 3 independent experiments, with the bar data indicating mean ± SD. Noting that there is no significant difference between the G_3⊂CB[7]@SC4A4/SP group and the G_3⊂CB[7]@SC4A4/MC group under each concentration for A549 cells.

Table S1. The phosphorescent lifetimes of different samples (the average lifetime: $\tau = \tau_1 \times Rel_1\% + \tau_2 \times Rel_2\%$).

Sample	Lifetime (%contribution)			
	τ_1 (μs)	τ_2 (μs)	τ	χ^2
G_3⊂CB[7] (540 nm)	50.1 (100 %)	—	50.1 μs	1.395
G_3⊂CB[7] + N_2 (540 nm)	648.4 (100 %)	—	0.648 ms	1.407
G_3⊂CB[7]@SC4A4 (540 nm)	716.1 (43.74%)	2639 (56.26%)	1.80 ms	1.315
G_3⊂CB[7]@SC4A4 + N_2 (540 nm)	1070 (23.74%)	3606 (76.26%)	3.00 ms	1.309
G_3⊂CB[7]@SC4A4/SP (540 nm)	527.4 (44.4%)	2333 (55.6%)	1.53 ms	1.306
G_3⊂CB[7]@SC4A4/MC (540 nm)	152.0 (42.22%)	798.0 (57.78%)	0.525 ms	1.254
G_3⊂CB[7]@SC4A4/MC (635 nm)	4.47 (31.69%)	120.6 (68.31%)	83.8 μs	1.314

Table S2. The fluorescent lifetimes of different samples (the average lifetime: $\tau = \tau_1 \times Rel_1\% + \tau_2 \times Rel_2\%$).

**Sample**	**Lifetime (ns) (%contribution)**			
	τ_1	τ_2	τ	χ^2
G_3 (390 nm)	0.37 (51.28%)	2.59 (48.72%)	1.45 ns	1.366
G_3⊂CB[7] (390 nm)	1.50 (55.78%)	8.81 (44.22%)	4.73 ns	1.287
G_3⊂CB[7]@SC4A4 (390 nm)	1.44 (53.09%)	8.33 (46.91%)	4.57 ns	1.527
SP (635 nm)	0.64 (66.97%)	1.96 (33.03%)	1.08 ns	1.290

7. Reference

[1] J. Cui, V. D. Uzunova, D.-S. Guo, K. Wang, W. M. Nau, Y. Liu, *Eur. J. Org. Chem.* **2010**, *9*, 1704-1710.

[2] J. Kim, H. Yun, Y. J. Lee, J. Lee, S.-H. Kim, K. H. Ku, B. J. Kim, *J. Am. Chem. Soc.* **2021**, *143* (33), 13333-13341.
